# Supplementary material for: Soil extracellular DNA fragments show variable degradation rates among sequences and environmental conditions
Source: eLife. 2026 Sep 4;15:RP110251. doi: 10.7554/eLife.110251 (PMC13544911; doi:10.7554/eLife.110251)
Supplement: Supplementary file 2 [file elife-110251-supp1.docx]

**Table A.** The geographic and climate information of the sampling sites included in this study.

| Sites | Ecosystems | Latitude (°N) | Longitude (°E) | MAT (℃) | MAP (mm) | Altitude (m) |
| --- | --- | --- | --- | --- | --- | --- |
| S1 | Grassland | 44.186 | 116.465 | 1.86 | 279 | 1083 |
| S2 | Grassland | 41.246 | 111.206 | 2.89 | 300 | 1715 |
| S3 | Grassland | 50.168 | 119.385 | -2.08 | 357 | 524 |
| S4 | Grassland | 37.614 | 101.310 | -0.7 | 469 | 3195 |
| S5 | Grassland | 31.248 | 92.079 | -1.26 | 453 | 4596 |
| S6 | Grassland | 31.274 | 92.111 | -0.24 | 445 | 4486 |
| S7 | Grassland | 30.831 | 91.146 | -1.5 | 418 | 4793 |
| S8 | Grassland | 31.440 | 90.030 | -0.66 | 423 | 4732 |
| S9 | Grassland | 34.838 | 98.324 | -3.35 | 340 | 4215 |
| S10 | Grassland | 36.113 | 100.451 | 3.17 | 364 | 2954 |
| S11 | Desert | 44.291 | 87.923 | 7.46 | 173 | 475 |
| S12 | Desert | 37.016 | 80.724 | 12.83 | 39 | 1362 |
| S13 | Desert | 39.494 | 110.191 | 6.55 | 371 | 1296 |
| S14 | Desert | 37.014 | 80.728 | 12.8 | 40 | 1364 |
| S15 | Cropland | 43.533 | 124.817 | 4.97 | 596 | 203 |
| S16 | Cropland | 36.831 | 116.562 | 13.14 | 605 | 21 |
| S17 | Cropland | 26.762 | 111.863 | 18.14 | 1373 | 117 |
| S18 | Cropland | 30.378 | 112.622 | 16.64 | 1094 | 27 |
| S19 | Cropland | 31.271 | 105.455 | 16.54 | 1001 | 444 |
| S20 | Cropland | 44.296 | 87.931 | 7.47 | 168 | 473 |
| S21 | Forest | 40.420 | 116.661 | 10.79 | 507 | 164 |
| S22 | Forest | 29.800 | 121.783 | 15.8 | 1288 | 170 |
| S23 | Forest | 33.588 | 108.876 | 11.68 | 734 | 732 |
| S24 | Forest | 21.917 | 101.167 | 21.69 | 1626 | 702 |
| S25 | Forest | 36.256 | 108.073 | 8.85 | 499 | 1348 |
| S26 | Forest | 23.052 | 112.473 | 22.53 | 1809 | 13 |
| S27 | Forest | 26.086 | 119.286 | 20.58 | 1382 | 15 |
| S28 | Forest | 35.586 | 118.257 | 12.77 | 778 | 163 |
| S29 | Forest | 38.454 | 106.267 | 8.95 | 204 | 1112 |
| S30 | Forest | 23.381 | 99.421 | 21.41 | 1429 | 991 |

MAT: mean annual temperature and MAP: mean annual precipitation.

**Table B.** The soil properties of the sampling sites included and the amount of exogenous DNA added in this study.

| Sites | pH | NO_3_^-^-N (mg kg^-1^) | NH_4_^+^-N (mg kg^-1^) | Moisture  (%) | TN  (g/kg) | TP (g/kg) | TOC  (g/kg) | AP  (mg kg^-1^) | Amount  (ng) |
| --- | --- | --- | --- | --- | --- | --- | --- | --- | --- |
| S1 | 7.26 | 19.00 | 3.17 | 6.49 | 1.72 | 0.39 | 15.76 | 22.50 | 747.5 |
| S2 | 6.69 | 10.47 | 3.73 | 3.67 | 1.45 | 0.36 | 14.92 | 12.71 | 480 |
| S3 | 6.71 | 14.30 | 7.53 | 7.31 | 2.76 | 0.50 | 33.12 | 20.61 | 1442.5 |
| S4 | 6.55 | 55.85 | 3.27 | 35.67 | 4.19 | 0.74 | 44.50 | 26.35 | 2835 |
| S5 | 6.72 | 58.82 | 2.88 | 29.21 | 4.36 | 0.71 | 51.28 | 18.09 | 1682.5 |
| S6 | 6.69 | 53.09 | 2.71 | 27.71 | 4.07 | 0.58 | 47.81 | 14.50 | 1250 |
| S7 | 6.42 | 44.72 | 3.04 | 10.42 | 2.70 | 0.53 | 28.09 | 14.14 | 857.5 |
| S8 | 6.21 | 77.65 | 2.55 | 12.8 | 2.37 | 0.47 | 22.05 | 23.12 | 532.5 |
| S9 | 8.85 | 10.03 | 2.17 | 7.60 | 0.78 | 0.36 | 7.41 | 35.94 | 417.5 |
| S10 | 8.16 | 5.96 | 3.22 | 6.51 | 0.33 | 0.27 | 4.28 | 18.14 | 265 |
| S11 | 7.83 | 30.12 | 3.42 | 2.89 | 0.66 | 0.97 | 7.25 | 62.48 | 245 |
| S12 | 8.31 | 19.56 | 2.76 | 1.29 | 0.14 | 0.50 | 2.27 | 12.76 | 197.5 |
| S13 | 8.34 | 3.58 | 2.96 | 0.76 | 0.54 | 0.45 | 10.38 | 84.64 | 200 |
| S14 | 8.07 | 22.89 | 10.96 | 0.77 | 0.24 | 0.61 | 3.75 | 85.99 | 255 |
| S15 | 5.98 | 17.93 | 2.88 | 14.83 | 1.13 | 0.39 | 18.57 | 57.94 | 535 |
| S16 | 6.14 | 28.99 | 2.55 | 15.16 | 1.17 | 1.00 | 10.79 | 42.66 | 1110 |
| S17 | 6.03 | 52.11 | 4.12 | 23.41 | 1.99 | 0.56 | 21.83 | 28.50 | 1045 |
| S18 | 6.23 | 43.84 | 2.81 | 18.59 | 1.60 | 0.74 | 17.96 | 35.32 | 495 |
| S19 | 6.98 | 21.37 | 3.94 | 15.72 | 0.68 | 0.51 | 8.49 | 24.19 | 314 |
| S20 | 6.73 | 14.18 | 2.73 | 10.60 | 0.71 | 1.18 | 7.19 | 81.96 | 475 |
| S21 | 7.10 | 14.74 | 4.35 | 18.88 | 1.10 | 0.42 | 22.45 | 19.15 | 897.5 |
| S22 | 6.84 | 7.19 | 4.14 | 22.48 | 0.43 | 0.18 | 5.34 | 9.74 | 275 |
| S23 | 7.33 | 137.54 | 3.58 | 35.22 | 4.73 | 0.90 | 58.93 | 26.87 | 2737.5 |
| S24 | 6.12 | 61.38 | 55.55 | 26.29 | 2.33 | 0.37 | 22.89 | 29.58 | 1352.5 |
| S25 | 7.70 | 58.99 | 2.65 | 15.65 | 1.33 | 1.15 | 11.74 | 29.56 | 957.5 |
| S26 | 4.15 | 15.94 | 34.82 | 16.54 | 2.13 | 0.28 | 30.01 | 15.58 | 632.5 |
| S27 | 5.46 | 56.22 | 2.73 | 29.77 | 1.97 | 1.15 | 23.72 | 13.78 | 1197.5 |
| S28 | 5.22 | 63.38 | 18.82 | 16.03 | 0.99 | 0.43 | 8.11 | 149.84 | 292.5 |
| S29 | 4.81 | 147.32 | 2.81 | 48.78 | 3.69 | 0.62 | 54.98 | 43.58 | 905 |
| S30 | 4.98 | 22.94 | 2.88 | 28.82 | 0.99 | 0.32 | 10.50 | 18.45 | 410 |

NO_3_^-^-N: soil NO_3_^-^-N contents; NH_4_^+^-N: soil NH_4_^+^-N contents; TN: soil total N contents; TP: soil total P contents; AP: soil available P contents; and TOC: soil total organic carbon contents.
