# Supplementary material for: Soil extracellular DNA fragments show variable degradation rates among sequences and environmental conditions
Source: eLife. 2026 Sep 4;15:RP110251. doi: 10.7554/eLife.110251 (PMC13544911; doi:10.7554/eLife.110251)
Supplement: Peer review history

# Editor report: eLife Assessment

**DOI:** 10.7554/eLife.110251.3.sa0

**Authors:** María Mercedes Zambrano

**Affiliations:** CorpoGen Colombia

**Keywords:** Convincing, Valuable

This **valuable** study introduces an innovative experimental design to address a crucial and timely issue in microbial ecology: the potential bias in soil microbial community analyses caused by extracellular DNA degradation. The work contributes to the field by providing **convincing** evidence that variable extracellular DNA degradation rates impact microbial ecology studies. This research will appeal to microbial ecologists and researchers interested in using molecular techniques to evaluate microbial community structure.

# Referee report: Reviewer #1 (Public review)

**DOI:** 10.7554/eLife.110251.3.sa1

Summary:

This manuscript investigates the degradation dynamics of extracellular DNA in soils and its impact on estimates of microbial abundance and diversity. By combining a broad geographic sampling design with a primer-labeling strategy, qPCR quantification, amplicon sequencing, and PMA treatment, the authors aim to disentangle total versus intracellular DNA signals and explore sequence-specific degradation patterns. The topic is relevant, particularly given the increasing awareness of relic DNA as a confounding factor in microbial ecology. The experimental design is ambitious and potentially impactful. However, several conceptual inconsistencies, methodological ambiguities, and statistical limitations currently weaken the robustness of the conclusions. These issues need to be addressed.

Strengths:

The manuscript addresses a timely and important question in microbial ecology, particularly given the growing recognition that relic DNA can bias interpretations of community composition derived from amplicon sequencing. The study is ambitious in scope, incorporating a broad geographic sampling design across multiple soil types, which enhances the generalizability of the findings. The use of a controlled microcosm experiment combined with a primer-labeling strategy to track extracellular DNA dynamics is conceptually innovative and provides a structured framework to investigate degradation processes.

In addition, the integration of multiple approaches, including qPCR for absolute quantification, high-throughput sequencing for community profiling, and PMA treatment to differentiate extracellular from intracellular DNA, represents a comprehensive attempt to disentangle complex sources of bias in soil microbiome analyses. The effort to link degradation dynamics with environmental variables and to explore sequence-level patterns further demonstrates the authors' intent to move beyond descriptive analyses toward a mechanistic understanding.

Weaknesses:

Several conceptual and methodological issues currently limit confidence in the study's conclusions. Key terms such as "sequence-specific degradation" are not clearly defined or supported by a mechanistic or structural hypothesis, making it difficult to interpret the biological meaning of the results. In addition, the bioinformatic workflow presents inconsistencies, particularly the use of ASVs followed by clustering at 97% similarity, which undermines the resolution required to support sequence-level inferences. Statistical analyses are also insufficiently described, including unclear definitions of "T values," a lack of detail on pairing structure, and no indication of multiple testing correction.

Furthermore, important methodological details are missing or unclear, including primer design (e.g., GAPDH tag vs ACTF), Illumina library preparation (e.g., adapter and indexing strategy), and validation of PMA treatment efficiency. The interpretation of PMA-treated samples as representing "living communities" is likely overstated, given the known limitations of the method in soil systems. Finally, typographical errors, inconsistent terminology, and unclear phrasing throughout the manuscript reduce readability and further complicate interpretation.

# Referee report: Reviewer #2 (Public review)

**DOI:** 10.7554/eLife.110251.3.sa2

Summary:

This manuscript describes the results of an interesting study examining the rate of degradation of extracellular DNA in soil ecosystems using a clever experimental approach. 16S ribosomal RNA genes were amplified from soil samples, and then purified PCR amplicons, containing a 5' linker sequence on the forward primer, were introduced to soils and monitored over time using real-time quantitative PCR and NGS amplicon sequencing. The study was able to measure rates of overall extracellular DNA degradation, but also sequence-specific degradation rates. I like the idea and execution of the study, and the results are interesting. The manuscript needs some help to improve the overall readability. Please see general and editorial comments below.

Strengths:

Innovative experimental design that is well deployed across a large number of soil types, revealing interesting variability in extracellular DNA degradation.

Weaknesses:

(1) The manuscript needs another review to improve the readability of the document.

(2) The authors have used 16S genes to look at sequence-specific degradation. But 16S rRNA genes are actually pretty well conserved, and there isn't as much genetic variation across this gene among organisms as there is for other genes. It might be more relevant to look at metagenomic DNA degradation from high AT, high GC organisms, etc. This would be more generalizable than 16S genes.

(3) Consideration of differential cell lysis during soil DNA extraction needs to be considered as well.

(4) It is not clear why the authors didn't put GAPDH linkers on the reverse primer as well. This would have given an easier amplicon to amplify (no degeneracies at all).

# Author comment: Author response

**DOI:** 10.7554/eLife.110251.3.sa3

**Authors:** Ting Li, Song Zhang, Zelin Wang, Wei Huang, Zejin Zhang, Fang Wang, Dong Liu, Xiaoyong Cui, Rongxiao Che

**Affiliations:** University of Chinese Academy of Sciences Beijing China; Nanjing University nanjing China; Yunnan University Kunming China; Yunnan University Kunming China; University of Chinese Academy of Sciences beijing China; University of Chinese Academy of Sciences beijing China; Yunnan University Kunming China; University of Chinese Academy of Sciences Beijing China; Yunnan University Kunming China

The following is the authors’ response to the original reviews.

We sincerely appreciate you and the reviewers for investing time and effort in evaluating our manuscript. After carefully reading the comments and suggestions, we found they are insightful, constructive, and critical for improving the quality of our work. Based on these valuable recommendations, we have substantially revised the manuscript as summarized below.

Abstract: Inappropriate or ambiguous statements have been revised to improve clarity.

Introduction: (1) The study purpose and hypotheses have been re-organized in a clearer and more concise way. (2) A mechanistic rationale for sequence-specific degradation has been provided and the use of PMA treatment has been explained. (3) The terminologies related to extracellular DNA and 16S rRNA gene amplicons have been clarified.

Materials and Methods: (1) More detailed description of the microcosm experiment has been added. (2) The design and rationale of GAPDH F-tagged primers and the use of fusion primers for Illumina library preparation have been clarified. (3) More details about PCR amplification, DNA purification, and pooling strategies have been added. (4) We have corrected and standardized primer naming throughout the manuscript; (5) More details about bioinformatic workflow have been added. (6) We have defined statistical parameters and multiple testing corrections. (7) All abbreviations have been defined and standardized.

Results: (1) The terminology for PMA-treated DNA has been revised and it has been clarified interpretation as “PMA-treated prokaryotic community” rather than “living community”. (2) The figures and legends have been updated for clarity, and the explicit explanation of “ASV I” and “ASV II” in pairwise comparisons have been added. (3) the figures (e.g., Figs. 2–5, S2–S8) have been reorganized to better reflect results; (4) Inappropriate statements or misleading interpretations have been removed.

Discussion: A detailed section on technical limitations have been added. The limiatons added mainly include: (1) PCR amplification bias and recommendations for spike-in standards or multi-primer approaches; (2) differential DNA extraction efficiency due to variable cell lysis; and (3) limitations of using 16S rRNA amplicons as proxies for natural extracellular DNA and the limitations of PMA treatment efficiency in soil matrices;

**eLife Assessment**

This valuable study introduces an innovative experimental design to address a crucial and timely issue in microbial ecology: the potential bias in soil microbial community analyses caused by extracellular DNA degradation. While the evidence showing variable degradation rates of extracellular DNA is convincing, additional conceptual, methodological, and statistical clarifications could reinforce the claims and the study's contribution to the field. This research will appeal to microbial ecologists and researchers interested in using molecular techniques to evaluate microbial community structure.

We sincerely appreciate the editors for the careful assessment of our work and for recognizing the value of addressing extracellular DNA degradation in soil microbial community analyses. We also greatly appreciate the reviewers’ constructive feedbacks concerning the need for additional conceptual, methodological, and statistical clarifications. We agree that further refinement in these areas will strengthen our claims and enhance the study’s contribution to the field. Based on these insightful suggestions, we have carefully revised the manuscript to provide clearer conceptual framework, more detailed methodological descriptions, and more rigorous statistical analyses. We believe these revisions have substantially improved the clarity and robustness of our work. More details about the revisions have been provided in the following responses.

**Public Reviews:**

**Reviewer #1 (Public review):**

Summary:

This manuscript investigates the degradation dynamics of extracellular DNA in soils and its impact on estimates of microbial abundance and diversity. By combining a broad geographic sampling design with a primer-labeling strategy, qPCR quantification, amplicon sequencing, and PMA treatment, the authors aim to disentangle total versus intracellular DNA signals and explore sequence-specific degradation patterns. The topic is relevant, particularly given the increasing awareness of relic DNA as a confounding factor in microbial ecology. The experimental design is ambitious and potentially impactful. However, several conceptual inconsistencies, methodological ambiguities, and statistical limitations currently weaken the robustness of the conclusions. These issues need to be addressed.

We sincerely appreciate the reviewer for the constructive assessment of our work. We also appreciate the reviewer’s critical insights regarding the conceptual inconsistencies, methodological ambiguities, and statistical limitations that currently weaken the robustness of the conclusions. We agree with the reviewer that addressing these issues is essential to strengthen our work. Based on these valuable comments, we have carefully revised the manuscript to clarify the conceptual framework. Additionally, we have provided more detailed methodological descriptions, and enhance the statistical rigor of our analyses. We believe these revisions have substantially improved the clarity, consistency, and overall robustness of our conclusions.

Strengths:

The manuscript addresses a timely and important question in microbial ecology, particularly given the growing recognition that relic DNA can bias interpretations of community composition derived from amplicon sequencing. The study is ambitious in scope, incorporating a broad geographic sampling design across multiple soil types, which enhances the generalizability of the findings. The use of a controlled microcosm experiment combined with a primer-labeling strategy to track extracellular DNA dynamics is conceptually innovative and provides a structured framework to investigate degradation processes.

In addition, the integration of multiple approaches, including qPCR for absolute quantification, high-throughput sequencing for community profiling, and PMA treatment to differentiate extracellular from intracellular DNA, represents a comprehensive attempt to disentangle complex sources of bias in soil microbiome analyses. The effort to link degradation dynamics with environmental variables and to explore sequence-level patterns further demonstrates the authors' intent to move beyond descriptive analyses toward a mechanistic understanding.

We sincerely thank the reviewer for the positive and encouraging comments of our work.

Weaknesses:

Several conceptual and methodological issues currently limit confidence in the study's conclusions. Key terms such as "sequence-specific degradation" are not clearly defined or supported by a mechanistic or structural hypothesis, making it difficult to interpret the biological meaning of the results. In addition, the bioinformatic workflow presents inconsistencies, particularly the use of ASVs followed by clustering at 97% similarity, which undermines the resolution required to support sequence-level inferences. Statistical analyses are also insufficiently described, including unclear definitions of "T values," a lack of detail on pairing structure, and no indication of multiple testing correction.

Furthermore, important methodological details are missing or unclear, including primer design (e.g., GAPDH tag vs ACTF), Illumina library preparation (e.g., adapter and indexing strategy), and validation of PMA treatment efficiency. The interpretation of PMA-treated samples as representing "living communities" is likely overstated, given the known limitations of the method in soil systems. Finally, typographical errors, inconsistent terminology, and unclear phrasing throughout the manuscript reduce readability and further complicate interpretation.

We sincerely appreciate the reviewer’s thorough and critical evaluation of the manuscript’s weaknesses. The issues raised regarding conceptual clarity, bioinformatic consistency, statistical rigor, methodological transparency, and the interpretation of PMA treatment have been fully acknowledged. We also recognized that typographical errors, inconsistent terminologies, and unclear phrasing largely reduced readability. In response to these valuable comments, the manuscript has been carefully revised as follows. (1) The clearer definition of the term “sequence-specific degradation” has been provided. (2) The bioinformatic workflow was streamlined to ensure consistency. (3) The descriptions of statistical analyses were substantially expanded, including explicit definitions of “t values,” detailed clarification of the pairing structure, and the application of appropriate multiple testing corrections. (4) Missing details regarding primer design, Illumina library preparation, and PMA treatment validation have been added to the Method section. (5) Interpretations of PMA-treated samples have been revised to more accurately reflect methodological limitations in soil systems. (6) The manuscript has been thoroughly proofread to correct typographical errors, standardize terminology, and enhance overall clarity. These revisions are believed to substantially address the concerns raised and significantly strengthen the manuscript. A point-by-point response to the specific comments is provided below.

**Reviewer #2 (Public review):**

Summary:

This manuscript describes the results of an interesting study examining the rate of degradation of extracellular DNA in soil ecosystems using a clever experimental approach. 16S ribosomal RNA genes were amplified from soil samples, and then purified PCR amplicons, containing a 5' linker sequence on the forward primer, were introduced to soils and monitored over time using real-time quantitative PCR and NGS amplicon sequencing. The study was able to measure rates of overall extracellular DNA degradation, but also sequence-specific degradation rates. I like the idea and execution of the study, and the results are interesting. The manuscript needs some help to improve the overall readability. Please see general and editorial comments below.

We sincerely thank the reviewer for the positive and encouraging assessment of our study. We have carefully revised the manuscript to enhance clarity, streamline the presentation, and refine the language throughout. We believe these improvements have made the manuscript more readable and easier to follow. We are also grateful for the general and editorial comments provided, which have been addressed as outlined below.

Strengths:

Innovative experimental design that is well deployed across a large number of soil types, revealing interesting variability in extracellular DNA degradation.

We sincerely thank the reviewer for the positive and encouraging assessment of our work.

Weaknesses:

(1) The manuscript needs another review to improve the readability of the document.

We thank the reviewer for this helpful suggestion. We fully agree that improving readability is essential for effectively communicating our findings. Based on the comment, we have carefully revised the manuscript to enhance clarity and readability. We have streamlined sentence structures, standardized terminology, corrected typographical errors, and improved the logical organization of the text. We believe these revisions have substantially improved the overall readability of the manuscript.

(2) The authors have used 16S genes to look at sequence-specific degradation. But 16S rRNA genes are actually pretty well conserved, and there isn't as much genetic variation across this gene among organisms as there is for other genes. It might be more relevant to look at metagenomic DNA degradation from high AT, high GC organisms, etc. This would be more generalizable than 16S genes.

We thank the reviewer for this insightful comment. We agree with the reviewer that 16S rRNA genes are relatively conserved compared to functional genes or whole metagenomic DNA, and that studying degradation of more variable sequences (e.g., high‑AT, high‑GC regions, or metagenomic DNA) would provide greater generalizability. However, we would like to clarify the rationale for using 16S rRNA gene amplicons in the present study. First, the 16S rRNA gene remains the most widely used phylogenetic marker in soil microbial ecology (Knight et al., 2018). Demonstrating sequence‑specific degradation with this well‑established marker directly informs a large body of existing research that relies on 16S RNA gene‑based community analyses. Second, despite its conserved nature, the targeted fragment in this study is belong to the highly varied region (V4) of 16S rRNA gene. Accordingly, we indeed observed significant sequence‑specific variation in degradation rates among different 16S rRNA gene amplicon sequence variants (ASVs) (Fig. 2c, 3a). This indicates that even within a conserved marker gene, sequence‑dependent degradation biases exist and can affect diversity estimates. Third, our study was designed as a proof‑of‑concept to establish a methodological framework for quantifying both overall and sequence‑specific degradation rates. Using a single, well‑characterized marker allowed us to develop and validate the primer‑labeling and qPCR/sequencing workflow without the additional complexity of metagenomic DNA (e.g., variable fragment lengths and complex mineral associations). In the revised manuscript, we have added the following sentence to the Discussion section to address the concerns from the reviewer.

L294-305

“Despite the high-resolution insights afforded by our methodology, several limitations should be considered. First, utilizing PCR-amplified 16S rRNA gene fragments as proxies oversimplifies the structural and sequence complexity of natural soil eDNA pools. In natural environments, eDNA varies widely in fragment length and conformation, and exhibits complex interactions with mineral surfaces, all of which fundamentally affect degradation dynamics (Levy-Booth et al., 2007; McKinney and Dungan, 2020). Additionally, the highly conserved nature of the 16S rRNA gene means that the nucleotide variability explored here (e.g., GC content gradients) does not fully capture the genomic heterogeneity of entire metagenomes (Knight et al., 2018). Consequently, our reported degradation rates indicate the decay potential of highly accessible linear eDNA rather than a universal rate for all soil DNA fractions. Future studies incorporating diverse metagenomic DNA, especially those with extreme AT or GC contents, are essential for building a more generalizable predictive framework for eDNA persistence (Morrissey et al., 2015)”

(3) Consideration of differential cell lysis during soil DNA extraction needs to be considered as well.

We thank the reviewer for raising this important technical consideration. We agree that differential cell lysis during soil DNA extraction is a well‑recognized source of bias in microbial community analysis. Different microbial taxa (e.g., Gram‑positive vs. Gram‑negative bacteria, spores, or fungi) vary in their cell wall structure and susceptibility to lysis, which can lead to under‑representation of certain groups and over‑representation of others in the extracted DNA. This bias affects both total DNA extracts and PMA‑treated fractions, potentially influencing our estimates of the relative contributions of intact‑cell derived DNA versus extracellular DNA. However, currently, eliminating these biases are still challenging, and thus we have added the following sentence to the Discussion to address this concern.

L305-311

“Second, methodological biases inherent in quantifying the intracellular community must be acknowledged (Du et al., 2025). Although PMA treatment is widely used to exclude eDNA, its efficiency in complex soil matrices can be compromised by limited light penetration in turbid suspensions and competitive adsorption to soil particles (Nocker et al., 2007; Carini et al., 2016; Heise et al., 2016). Compounding this issue, downstream DNA recovery is subject to differential cell lysis, as taxa with robust cell walls (e.g., Gram-positive bacteria) may resist extraction (Frostegård et al., 1999; Albertsen et al., 2015).”

(4) It is not clear why the authors didn't put GAPDH linkers on the reverse primer as well. This would have given an easier amplicon to amplify (no degeneracies at all).

The decision to place the GAPDH linker only on the forward primer (515F) was intentional to balance the need for tracking exogenous extracellular DNA with amplification efficiency, sequencing quality, and cost-effectiveness. Adding linkers to both primers would increase the total amplicon length, potentially reducing amplification efficiency, especially in complex soil samples with degraded or low-quality DNA. More importantly, the reverse primer used in our study is a degenerate primer designed to target the 16S rRNA gene across diverse bacterial taxa, and extending it with an additional GAPDH linker could introduce further complexity, decrease amplification efficiency, and increase primer-dimer formation. Additionally, single-end labeling allows the usage of standard 16S rRNA reverse primers with existing barcodes, whereas dual-end labeling would require synthesis of new barcode-labeled primers, increasing both cost and time. Our preliminary experiments confirmed that single-end labeling produced reproducible amplification curves (~85% efficiency) and high-quality sequencing reads, which were sufficient for quantifying degradation rates. We have added a clarification in the Methods section to explain this rationale.

L365-371

“The GAPDH was incorporated only into the forward primer for several reasons. Methodologically, adding a long linker to the degenerate reverse primer (806R) could reduce amplification efficiency or introduce bias. Economically, single-end labeling allowed us to use the standard reverse primer already carrying sample-specific barcodes, avoiding the costly synthesis of a full set of dual-labeled barcoded primers. This design minimized the risk of secondary structure and primer-dimer artifacts while maintaining sufficient specificity and compatibility with downstream qPCR and sequencing.”

**Recommendations for the authors:**

**Reviewer #1 (Recommendations for the authors):**

Major Comments

(1) Inconsistency between ASV inference and 97% sequence recruitment

The bioinformatic pipeline presents a major conceptual inconsistency. ASVs are inferred using UNOISE3, but reads are subsequently mapped to ASVs at a 97% similarity threshold, effectively reintroducing OTU-level clustering. Given that the manuscript's central claim is sequence-specific degradation, this step undermines the single-nucleotide resolution that ASVs provide and may obscure biologically meaningful differences. The authors should either reanalyze the data using a consistent ASV framework (exact matching), or explicitly treat the analysis as OTU-like and moderate claims of sequence specificity.

We appreciate the reviewer's critical evaluation of our bioinformatic pipeline. We also apologized for our unclear statements in our original manuscript. We understand the concern that mapping reads to ASVs at 97% similarity might appear to reintroduce OTU-level clustering. Actually, we used the default pipeline provided by the authors of USEARCH with otutab command to generate the ASV table.

Following the logic and recommendations of the USEARCH/UNOISE3 developer (Robert Edgar), this approach is a standard procedure for robust noise management rather than a conceptual inconsistency. First, in our pipeline, ASVs (ZOTUs) are first inferred using the UNOISE3 algorithm, which effectively identifies “true” biological sequences at single-nucleotide resolution. Secondly, according to the USEARCH manual, while the ASVs themselves represent exact biological sequences, the raw reads inevitably contain stochastic sequencing errors. Using “exact matching” for recruitment would discard a significant portion of the data that originates from a specific ASV but carries minor random errors. Mapping at 97% identity is the recommended method to recruit these noisy reads back to their correct biological origin (the ASV centroid). Meanwhile, during the recruitment process, reads are not randomly assigned to any ASV within the 97% identity radius. Instead, the algorithm follows a “highest similarity first” principle. For instance, if a specific read exhibits 98% similarity to ASV1 and 97% similarity to ASV2, it is strictly assigned to ASV1. A read is only discarded if its highest similarity to any ASV falls below the 97% threshold. Unlike traditional OTU clustering (where sequences are clustered together based on similarity from the start), our approach maintains the ASV as a fixed biological reference. The quantification of degradation rates is performed on these high-resolution centroids. Thus, our claims regarding sequence-specific degradation remain valid, as the underlying biological variation is defined by the ASVs. To avoid any possible confusion, we have rewritten the relevant paragraph in the Methods section (subsection 4.6) as follows.

L446-457

“ASVs were generated using the UNOISE3 non‑clustering denoising algorithm (Edgar, 2016), which infers 100% exact sequence variants by distinguishing biological sequences from PCR/sequencing errors. ASVs with total sequence counts fewer than 9 across all samples were removed to reduce noise. To quantify the abundance of each ASV, an ASV table was generated by mapping the quality‑filtered raw reads back to the ASV set using the otutab command. A 97% similarity threshold was applied for this recruitment to accommodate stochastic sequencing noise while maintaining biological resolution. Crucially, the mapping followed a best-hit priority rule, where each read was assigned to the ASV with the highest per cent identity within the 97% radius. This approach ensures that reads derived from the same biological template are accurately counted toward their respective ASV, preventing the underestimation of abundances that would occur with exact matching while strictly preserving the single-nucleotide resolution of the ASV framework.”

(2) Undefined "ASV I" and "ASV II" groups

The manuscript refers to "ASV I" and "ASV II" groups in pairwise comparisons of degradation rates (e.g., Fig. 3), but these groups are not defined anywhere in the text.

It is unclear whether these represent: predefined biological categories, arbitrary pairwise ASV comparisons, or groupings based on taxonomy, abundance, or degradation rate.

In addition, the pairing structure underlying these comparisons is not described. While a paired t-test is mentioned, it is unclear how ASVs were paired (e.g., within sites, across samples, or across time points).

The current terminology ("groups") is potentially misleading and suggests biological structure where none may exist. The authors should explicitly define these terms, clarify the pairing scheme, and revise terminology if these are simply pairwise comparisons.

We thank the reviewer for this keen observation. We completely agree that the terms "ASV I" and "ASV II" were poorly defined and potentially misleading.

We would like to clarify that "ASV I" and "ASV II" were not intended to represent predefined biological categories (such as groupings based on taxonomy, abundance, or degradation rates). Instead, they were merely used as a labeling convention to indicate the directionality of pairwise comparisons within the heatmap matrix. Specifically, "ASV I" referred to the ASVs represented in the rows, while "ASV II" referred to those in the columns. In the original Fig. 3, blue indicated that the degradation rate of the row ASV was significantly lower than that of the column ASV, and red indicated the opposite. To avoid any confusion, we have removed the “ASV I/II” terminology throughout the manuscript and figures, replacing them with “Row ASVs” and “Column ASVs”. To address this issue, we have revised the Figure 3 legend to include a more explicit explanation:

L820-824

“In the heatmap, each cell represents a pairwise comparison between two ASVs. Blue indicates that the degradation rate of the ASVs listed in the row (row ASVs) is significantly lower than that of the ASVs listed in the column (column ASVs); red indicates that the row ASVs has a significantly higher degradation rate than the column ASV. A positive t value indicates that the row ASVs degrades significantly faster than the column ASVs; a negative t value indicates the opposite.”

We thank the reviewer for raising the important issue regarding the definition of “t values” in our statistical analysis. We apologize for the lack of clarity in the original manuscript. To clarify, the T values presented in Figure 3a represent the test statistics (t-values) from paired t-tests comparing the degradation rate constants of two ASVs across the 30 study sites. The T-value was obtained from a paired t-test between two ASVs across the same samples. The t-value indicates the magnitude and direction of the difference between the two ASVs’ degradation rates relative to the variability across sites. A positive t-value (colored red in the heatmap) indicates the row ASVs degrades significantly faster than the column ASVs; a negative t-value (colored blue) indicates the opposite.

L544-548

“As for the analysis, we performed paired t‑tests across all the study sites. Thus, the degradation rates were essentially compared within each site, with both values originating from a same soil sample under identical incubation conditions. A positive t value indicates that the first ASV has a significantly higher degradation rate than the second one, and a negative t value indicates the opposite. The p values were adjusted for multiple comparisons using the FDR method.”

(3) Lack of definition and justification of "T values"

The manuscript reports "T values" for comparisons between ASVs but does not clearly define how these values are calculated. Although a paired t-test is mentioned, it remains unclear how the pairing was constructed, whether assumptions (normality, independence) were evaluated, and whether corrections for multiple comparisons were applied. Given the large number of ASVs, failure to control for multiple testing could inflate false positives. More broadly, the use of a simple paired t-test may not be appropriate given the hierarchical and compositional structure of the data.

We sincerely thank the reviewer for pointing out the need to clarify the definition and justification of the t-values presented in our manuscript. Each t-value represents the test statistic from a paired t-test comparing the degradation rates of two ASVs across the same set of samples. The paired t-test assumes that the differences between paired observations are approximately normally distributed and that the pairs are independent across columns. We have evaluated the normality of differences using standard diagnostic plots and verified that the assumption is reasonably satisfied given the sample size. We performed a correction for multiple comparisons using the False Discovery Rate (FDR) procedure to control for potential false positives. We have revised the Methods section to clearly define t-values.

(4) Conceptual validity of "sequence-specific degradation"

The manuscript repeatedly refers to "sequence-specific degradation" of extracellular DNA; however, this concept is not clearly defined nor supported by a biological or structural hypothesis. It is unclear what "sequence-specific" refers to (e.g., nucleotide composition, GC content, secondary structure, taxonomic identity), whether differences are expected in conserved versus variable regions of the 16S rRNA gene, or what mechanistic basis would explain differential degradation among sequences. Given that the analysis is based on short 16S V4 amplicons, and no structural or biochemical framework is provided, it is difficult to interpret whether the observed differences truly reflect intrinsic sequence-dependent degradation or are instead driven by methodological or statistical artifacts (e.g., abundance effects, amplification bias).

I believe the authors should explicitly define what is meant by "sequence-specific degradation," provide a biologically grounded hypothesis (e.g., structural accessibility, GC content, stem-loop stability), and align their interpretation with the resolution and limitations of the data.

We thank the reviewer for this critical conceptual comment. We apologize that “sequence‑specific degradation” was not clearly defined and lacked a biological or structural hypothesis. To improve the logical flow of the manuscript, we have restructured the Introduction by moving the three central hypotheses immediately following the discussion of the biochemical mechanisms underlying sequence-specific degradation. This adjustment ensures that the hypotheses are directly grounded in the theoretical framework (e.g., GC content, thermodynamic stability, and secondary structures) presented in the paragraph.

We now define “sequence‑specific degradation” as statistically significant differences in first‑order degradation rate constants among distinct ASVs, mainly arising from intrinsic DNA properties (base composition, secondary structure, and restriction sites) or differential mineral adsorption.

L99-104

“Consequently, we proposed three central hypotheses. (1) The degradation rates of eDNA amplicon fragments were expected to be highly sequence‑specific. (2) The rates and patterns of eDNA fragments degradation would be influenced by environmental factors such as temperature and moisture content. (3) The sequence‑specific degradation of extracellular 16S rRNA gene amplicon fragments would significantly influence estimates of soil prokaryotic abundance and diversity.”

We also expanded the mechanistic discussion to include GC content and secondary structure.

L230-235

“We also examined whether GC content could explain the observed sequence‑specific patterns, but no significant correlation was found (Fig. S4), suggesting that simple base composition is not the primary driver in this study. However, this does not exclude the possibility that higher‑order structural features (e.g., hairpin loops) or sequence‑specific nuclease recognition motifs contribute to differential degradation (Wang et al., 2007). This should be tested in future studies using synthetic DNA constructs with controlled structural elements.”

We acknowledge that inferring sequence‑specific degradation from combined relative abundance and qPCR data is subject to potential methodological artifacts, including compositional effects, PCR amplification bias, and abundance‑dependent detection limits. However, we have taken several stringent steps to minimize these concerns. Specifically, we restricted our analysis to ASVs that were present in more than 90% of the study sites and for which the degradation curve fits yielded R^2^ > 0.5, ensuring that only robustly detected and reliably modeled sequences were retained. Because our analysis tracks the ratio of each ASV across a time series, any sequence-specific PCR amplification bias remains constant for that particular sequence. By focusing on the rate of change rather than absolute read counts, such systematic biases are mathematically canceled out during the calculation of degradation kinetics.

(5) Conceptual ambiguity in "GAPDH F-labeled 16S rRNA genes"

The manuscript repeatedly refers to "GAPDH F-labeled 16S rRNA genes," which is confusing and may be misinterpreted as targeting GAPDH rather than 16S. It should be clearly stated that GAPDH refers to glyceraldehyde-3-phosphate dehydrogenase, and a GAPDH-derived sequence is used as a synthetic tag appended to a 16S primer. Additionally, the divergence of this tag from microbial sequences should be justified to ensure specificity. There is also an inconsistency in primer naming (e.g., "GAPDH F" vs "ACTF" in the figures), which should be corrected.

We sincerely thank the reviewer for this important comment. We agree that the phrase “GAPDH F‑labeled 16S rRNA genes” could be confusing, as it may be misinterpreted as targeting the GAPDH gene rather than the 16S rRNA gene. We have revised the manuscript to avoid this ambiguity and to provide clear justification for the use of the GAPDH tag. GAPDH (glyceraldehyde‑3‑phosphate dehydrogenase) is a human housekeeping gene. Its forward primer sequence (GAPDH F: 5′‑CAT TGG CAA TGA GCG GTT C‑3′) was used as a synthetic tag appended to the 16S primer because (i) no homologous sequences exist in soil DNA (confirmed by PCR), and (ii) its melting temperature is compatible with the reverse primer. This tag allows specific tracking of exogenous DNA without interference from native soil sequences.

Throughout the manuscript, ambiguous phrases such as “GAPDH F‑labeled 16S rRNA genes” have been replaced with more precise terms, “GAPDH F‑tagged 16S rRNA gene amplicon fragments” clarifying that the tag is an appendage and not the amplification target.

We have checked the entire manuscript and confirm that “ACTF” does not appear anywhere. To avoid confusion, the primer is now consistently referred to as “GAPDH F” in all figures, legends, and text.

L360-371

“GAPDH is a primer for a human housekeeping gene and it has no homologous sequences in soils. Subsequently, GAPDH was selected as the label primer based on two criteria. First, this primer was selected to avoid interference from the original soil sequences (Huang et al., 2014; Yang et al., 2021; Arvizu-Hernandez et al., 2025), and no detectable PCR amplification was observed for the primer set GAPDH F-806R across all the soil DNA samples included in this study. Second, the melting temperature (Tm) value of GAPDH F approximately matched that of 806R. The GAPDH was incorporated only into the forward primer for several reasons. Methodologically, adding a long linker to the degenerate reverse primer (806R) could reduce amplification efficiency or introduce bias. Economically, single-end labeling allowed us to use the standard reverse primer already carrying sample-specific barcodes, avoiding the costly synthesis of a full set of dual-labeled barcoded primers. This design minimized the risk of secondary structure and primer-dimer artifacts while maintaining sufficient specificity and compatibility with downstream qPCR and sequencing.”

(6) Limitations of using PCR amplicons as proxies for extracellular DNA

The study uses PCR-generated amplicons to simulate extracellular DNA. While useful for controlled comparisons, these fragments may not reflect the physicochemical diversity of natural extracellular DNA (e.g., adsorption to minerals, fragment size variability, protection within aggregates). This limitation should be explicitly acknowledged, and conclusions should be framed accordingly.

We appreciate the reviewer’s constructive feedback. We fully acknowledge that using PCR-generated amplicons to simulate extracellular DNA (eDNA) has inherent limitations in capturing the full physicochemical diversity of naturally occurring eDNA in soils. Specifically, we agree that PCR fragments may not replicate features such as highly variable fragment size distributions, associations with complex cellular components (e.g., vesicles or protein complexes), or long-term physical sequestration within soil micro-aggregates. Despite of these limitations, the use of uniform primer-tagged PCR amplicons was a deliberate choice to enable precise tracking of exogenous DNA degradation kinetics while eliminating background interference from endogenous soil eDNA. This design is a prerequisite for the high-resolution kinetic modeling of sequence-specific decay. Furthermore, in our bioinformatic pipeline, the 97% mapping threshold was specifically applied to minimize the influence of stochastic sequencing and PCR errors on abundance quantification. In the revised manuscript, these potential limitations have been addressed.

L295-299

“First, utilizing PCR-amplified 16S rRNA gene fragments as proxies oversimplifies the structural and sequence complexity of natural soil eDNA pools. In natural environments, eDNA varies widely in fragment length and conformation, and exhibits complex interactions with mineral surfaces, all of which fundamentally affect degradation dynamics (Levy-Booth et al., 2007; McKinney and Dungan, 2020).”

(7) Interpretation of sequence-specific degradation

Sequence-specific degradation rates are inferred from combining relative abundance data with total qPCR estimates. This approach is sensitive to compositional effects, amplification biases, and abundance-dependent detection limits. It remains unclear whether observed differences reflect true sequence-specific degradation or methodological artifacts. This limitation should be discussed more explicitly.

We thank the reviewer for highlighting this critical methodological point. In our study, sequence-specific degradation rates were estimated by combining ASV-relative abundances with total qPCR-derived 16S rRNA gene copy numbers. We acknowledge that this approach may be influenced by compositional effects, PCR amplification biases, and abundance-dependent detection limits. However, the degradation rate constant (*k*) in our study, represents the rate of change for a specific sequence over time. Since PCR amplification biases are generally sequence-specific and consistent across samples processed under identical conditions, these systematic errors are mathematically canceled out when calculating the relative change (slope) for the same ASV across a time series. Second, all qPCR measurements were performed with three technical triplicates with standard curves to ensure quantitative reliability. Third, relative abundances were converted to absolute abundances using total qPCR estimates, allowing cross-taxa comparisons that reduce compositional bias. This approach is widely recognized in microbial ecology as a robust method. To address this concern, we have added some explanations in the revised manuscript.

L84-86

“In this study, “sequence‑specific degradation” refers to statistically significant differences in first‑order degradation rate constants (k, day^⁻¹^) among distinct 16S rRNA gene amplicon sequence variants (ASVs) under identical soil and incubation conditions.”

L294-305

“Despite the high-resolution insights afforded by our methodology, several limitations should be considered. First, utilizing PCR-amplified 16S rRNA gene fragments as proxies oversimplifies the structural and sequence complexity of natural soil eDNA pools. In natural environments, eDNA varies widely in fragment length and conformation, and exhibits complex interactions with mineral surfaces, all of which fundamentally affect degradation dynamics (Levy-Booth et al., 2007; McKinney and Dungan, 2020). Additionally, the highly conserved nature of the 16S rRNA gene means that the nucleotide variability explored here (e.g., GC content gradients) does not fully capture the genomic heterogeneity of entire metagenomes (Knight et al., 2018). Consequently, our reported degradation rates indicate the decay potential of highly accessible linear eDNA rather than a universal rate for all soil DNA fractions. Future studies incorporating diverse metagenomic DNA, especially those with extreme AT or GC contents, are essential for building a more generalizable predictive framework for eDNA persistence (Morrissey et al., 2015).”

L311-314

“While our standardized bead-beating protocol and calculation of degradation rate constants (*k*) minimize systematic biases, future studies should integrate complementary viability markers (e.g., RNA-based analyses or protein synthesis activity probes) and multi-extraction comparisons to robustly validate these ecological patterns (Emerson et al., 2017)..”

(8) Overinterpretation of PMA-treated samples as "living communities"

The manuscript interprets PMA-treated DNA as representing intracellular or "living" microbial communities. While PMA is useful, this interpretation should be treated with caution in soils. PMA efficiency can be affected by soil matrix complexity, DNA adsorption to particles, incomplete light penetration, and permeability of compromised cells. Importantly, no validation of PMA efficiency is presented.

We thank the reviewer for this important caution. We agree that interpreting PMA‑treated DNA as representing “living” or “intracellular” communities is an overstatement in soil systems. In the revised manuscript, we no longer describe PMA-treated DNA as a direct proxy for the “living community,” but instead refer to it as the “PMA-treated prokaryotic community”.

Although we did not directly validate PMA efficiency in this study, we used a standardized PMA protocol that has been widely applied in microbial ecology, and our goal was to obtain a comparative estimate of the influence of extracellular DNA on community analysis across soils under a consistent methodological framework. Based on previous studies (Carini et al., 2016; Du et al., 2025), which found that in similar soil types, PMA treatment can significantly reduce the interference of extracellular DNA and alter the community structure, this indirectly proves the effectiveness of this technique.

Nevertheless, we agree that future studies should include explicit validation controls, such as live/dead cell mixtures, heat-killed controls, or soil-specific PMA efficiency tests, to better quantify method performance across diverse soil matrices. We have added a dedicated paragraph in the "Methodological Considerations and Limitations" section to discuss how soil-specific properties (e.g., turbidity, adsorption capacity) might lead to incomplete exclusion of extracellular DNA, thereby advising a more cautious interpretation of the "viable" community data.

L496-500

“To inhibit amplification of eDNA, soils were incubated with propidium monoazide (PMA), as described previously (Carini et al., 2016). Upon photoactivation, eDNA can form covalent bonds through cross-linking, leading to the inhibition of its PCR amplification. In contrast, microbes with intact cell membranes exclude PMA, and their DNA is not cross-linked with PMA, and remains amenable to PCR amplification.”

L294-305

“Despite the high-resolution insights afforded by our methodology, several limitations should be considered. First, utilizing PCR-amplified 16S rRNA gene fragments as proxies oversimplifies the structural and sequence complexity of natural soil eDNA pools. In natural environments, eDNA varies widely in fragment length and conformation, and exhibits complex interactions with mineral surfaces, all of which fundamentally affect degradation dynamics (Levy-Booth et al., 2007; McKinney and Dungan, 2020). Additionally, the highly conserved nature of the 16S rRNA gene means that the nucleotide variability explored here (e.g., GC content gradients) does not fully capture the genomic heterogeneity of entire metagenomes (Knight et al., 2018). Consequently, our reported degradation rates indicate the decay potential of highly accessible linear eDNA rather than a universal rate for all soil DNA fractions. Future studies incorporating diverse metagenomic DNA, especially those with extreme AT or GC contents, are essential for building a more generalizable predictive framework for eDNA persistence (Morrissey et al., 2015).”

Minor Comments

(1) Line 79: Provide examples of how extracellular DNA contributes to nutrient cycling (e.g., P, N sources) and signal transduction (e.g., horizontal gene transfer).

We thank the reviewer for this helpful suggestion. In the revised manuscript, we have added specific examples to clarify how extracellular DNA contributes to nutrient cycling and signal transduction. Specifically, we now note that extracellular DNA can serve as a source of phosphorus and nitrogen following enzymatic degradation, thereby contributing to soil nutrient turnover. We also clarify that extracellular DNA plays an important role in horizontal gene transfer, acting as a genetic reservoir that can be taken up by competent microorganisms and thereby facilitating the spread of functional traits such as antibiotic resistance. These examples have been added to improve the clarity and biological context of this statement.

L48-52

“EDNA serves as a critical vector for horizontal gene transfer (HGT), facilitating the uptake of genetic material by competent microorganisms and promoting the spread of functional traits such as antibiotic resistance (Liu et al., 2024). In addition, eDNA participates in soil biogeochemical cycling because its enzymatic degradation releases bioavailable nutrients, particularly phosphorus and nitrogen, which can be reused by soil microorganisms (Ye et al., 2022).”

(2) Line 79: Replace "for an extended period of time" with a more precise or referenced timescale.

We agree with the reviewer. We have replaced the vague phrase with a precise timescale. Extracellular DNA can persist in soils for months to years.

(3) Line 94: Clarify what is meant by "high-level structure" (e.g., secondary structure, environmental association).

We thank the reviewer for pointing out this ambiguity. In the original manuscript, the phrase “high-level structure” was not sufficiently precise. In the revised version, we have clarified that this refers primarily to higher-order structural properties of DNA molecules, such as secondary structure, local conformational features, and sequence-dependent interactions with minerals or organic matter in soil. These characteristics may influence the accessibility of extracellular DNA to nucleases and thus affect degradation rates. We have revised the text accordingly to improve clarity and precision.

L84-104

“In this study, “sequence‑specific degradation” refers to statistically significant differences in first‑order degradation rate constants (k, day^⁻¹^) among distinct 16S rRNA gene amplicon sequences (ASVs) under identical soil and incubation conditions. The potential variations in sequence-specific eDNA degradation rates can be attributed to several factors. First, sequence-dependent degradation can arise from differences in nucleotide composition, particularly GC content. This influences the thermodynamic stability and base-stacking interactions of the DNA duplex, thereby altering its accessibility to extracellular nucleases (Marrone and Ballantyne, 2008; Wolpe and Guertin, 2022). Second, local conformational features and the formation of potential secondary structures, such as stem-loops or hairpins, can create steric hindrance that protects the phosphodiester backbone. Differences in base composition also alter the elemental stoichiometry (e.g., C: N ratio) of DNA molecules, potentially affecting microbial preference for recycling specific sequences as nutrient sources (Cai et al., 2006a; Buitrago et al., 2021). Third, the persistence of soil DNA is often associated with its adsorption and protection by minerals and humus in soils (Cai et al., 2006b; Vuillemin et al., 2017; McKinney and Dungan, 2020). Thus, sequence-dependent differences in the physicochemical behavior of DNA molecules, including their affinity for soil minerals and organic matter, may also contribute to variation in degradation rates among sequences (Levy-Booth et al., 2007; Morrissey et al., 2015). Consequently, we proposed three central hypotheses. (1) The degradation rates of eDNA amplicon fragments were expected to be highly sequence‑specific. (2) The rates and patterns of eDNA fragments degradation would be influenced by environmental factors such as temperature and moisture content. (3) The sequence‑specific degradation of extracellular 16S rRNA gene amplicon fragments would significantly influence estimates of soil prokaryotic abundance and diversity.”

(4) Line 111: The hypothesis is not clearly linked to the rationale. If sequence-specific degradation is expected, clarify whether it relates to conserved vs variable regions or structural features (e.g., stems vs loops).

We thank the reviewer for this helpful comment. We agree that the original manuscript did not clearly link the hypothesis regarding sequence-specific degradation to its mechanistic rationale. In the revised manuscript, we have clarified that the expectation of sequence-specific degradation is not simply based on conserved vs variable regions of the 16S rRNA gene, but rather on the potential for sequence differences to influence intrinsic physicochemical properties, including base composition, local conformational features, potential secondary structures, and motif-dependent nuclease susceptibility. These factors may alter DNA accessibility to extracellular nucleases, providing a mechanistic basis for sequence-specific degradation. This clarification is now reflected in the Introduction and linked to the formal hypothesis statement.

To improve the logical flow of the manuscript, we have restructured the Introduction by moving the three central hypotheses (H1–H3) immediately following the discussion of the biochemical mechanisms underlying sequence-specific degradation.

L84-104

“In this study, “sequence‑specific degradation” refers to statistically significant differences in first‑order degradation rate constants (k, day^⁻¹^) among distinct 16S rRNA gene amplicon sequences (ASVs) under identical soil and incubation conditions. The potential variations in sequence-specific eDNA degradation rates can be attributed to several factors. First, sequence-dependent degradation can arise from differences in nucleotide composition, particularly GC content. This influences the thermodynamic stability and base-stacking interactions of the DNA duplex, thereby altering its accessibility to extracellular nucleases (Marrone and Ballantyne, 2008; Wolpe and Guertin, 2022). Second, local conformational features and the formation of potential secondary structures, such as stem-loops or hairpins, can create steric hindrance that protects the phosphodiester backbone. Differences in base composition also alter the elemental stoichiometry (e.g., C:N ratio) of DNA molecules, potentially affecting microbial preference for recycling specific sequences as nutrient sources (Cai et al., 2006a; Buitrago et al., 2021). Third, the persistence of soil DNA is often associated with its adsorption and protection by minerals and humus in soils (Cai et al., 2006b; Vuillemin et al., 2017; McKinney and Dungan, 2020). Thus, sequence-dependent differences in the physicochemical behavior of DNA molecules, including their affinity for soil minerals and organic matter, may also contribute to variation in degradation rates among sequences (Levy-Booth et al., 2007; Morrissey et al., 2015). Consequently, we proposed three central hypotheses. (1) The degradation rates of eDNA amplicon fragments were expected to be highly sequence‑specific. (2) The rates and patterns of eDNA fragments degradation would be influenced by environmental factors such as temperature and moisture content. (3) The sequence‑specific degradation of extracellular 16S rRNA gene amplicon fragments would significantly influence estimates of soil prokaryotic abundance and diversity.”

(5) Lines 310-311: Clearly indicate which portion of the primers corresponds to the modified (GAPDH-derived) sequence. Provide full annotated primer sequences.

We thank the reviewer for this helpful suggestion. In the revised manuscript, we now clearly indicate which portion of the forward primer corresponds to the GAPDH-derived synthetic tag and which portion corresponds to the 16S rRNA gene primer sequence. We have also provided the full annotated primer sequences in the Methods section to avoid ambiguity.

Specifically, the modified forward primer is now described as:

GAPDH-F-515F: 5′-CAT TGG CAA TGA GCG GTT C-GTG CCA GCM GCC GCG GTA A-3′,

where CAT TGG CAA TGA GCG GTT C is the GAPDH-derived synthetic tag and GTG CCA GCM GCC GCG GTA A is the 16S rRNA gene forward primer sequence (515F).

The reverse primer is:

806R: 5′-GGA CTA CHV GGG TWT CTA AT-3′.

L354-359

“Briefly, exogenous eDNA was prepared by PCR amplification using a modified forward primer consisting of a GAPDH F tag fused to the 16S rRNA gene primer 515F, together with the reverse primer 806R. The full primer sequences were as follows: GAPDH-F-515F: 5'-CAT TGG CAA TGA GCG GTT C-GTG CCA GCM GCC GCG GTA A-3', in which CAT TGG CAA TGA GCG GTT C represents the GAPDH F tag and GTG CCA GCM GCC GCG GTA A represents the 16S rRNA gene forward primer sequence (515F); and 806R: 5'-GGA CTA CHV GGG TWT CTA AT-3'.”

(6) Lines 310-311: Explicitly define GAPDH and justify its use as a synthetic tag.

We thank the reviewer for this helpful suggestion. In the revised manuscript, we now explicitly define GAPDH as glyceraldehyde-3-phosphate dehydrogenase, a human housekeeping gene. Specifically, the GAPDH-derived sequence was selected for two reasons. First, it is highly divergent from known soil microbial 16S rRNA gene sequences and did not produce detectable amplification when tested with soil DNA using the GAPDH tagged 806R primer pair, indicating that it would not interfere with endogenous soil DNA signals. Second, its melting temperature was compatible with that of the reverse primer, which allowed stable amplification of the tagged 16S amplicons under our PCR conditions.

L360-371

“GAPDH is a primer for a human housekeeping gene and it has no homologous sequences in soils. Subsequently, GAPDH was selected as the label primer based on two criteria. First, this primer was selected to avoid interference from the original soil sequences (Huang et al., 2014; Yang et al., 2021; Arvizu-Hernandez et al., 2025), and no detectable PCR amplification was observed for the primer set GAPDH F-806R across all the soil DNA samples included in this study. Second, the melting temperature (Tm) value of GAPDH F approximately matched that of 806R. The GAPDH was incorporated only into the forward primer for several reasons. Methodologically, adding a long linker to the degenerate reverse primer (806R) could reduce amplification efficiency or introduce bias. Economically, single-end labeling allowed us to use the standard reverse primer already carrying sample-specific barcodes, avoiding the costly synthesis of a full set of dual-labeled barcoded primers. This design minimized the risk of secondary structure and primer-dimer artifacts while maintaining sufficient specificity and compatibility with downstream qPCR and sequencing”

(7) Line 346: Start a new paragraph to clearly separate this as a distinct experiment.

We thank the reviewer for this helpful suggestion. In the revised manuscript, we have started a new paragraph.

(8) Line 346: Specify the number of samples analyzed for consistency.

We thank the reviewer for this helpful suggestion. In the revised manuscript, we have now explicitly specified the number of samples.

A total of 120 samples were analyzed in this moisture gradient experiment: 2 ecosystems (Kaiyuan and Dashanbao) × 5 moisture levels (10%, 25%, 50%, 75%, and 100% of water holding capacity) × 6 incubation time points (0, 1, 3, 6, 12, and 24 days) × 2 replicates. Just two technical replicates were performed for this validation experiment, as the primary aim was to assess the trend of moisture effects rather than statistical inference across replicates.

L408-410

“This complementary experiment included two sites, five moisture levels, six incubation time points, and two replicates per treatment combination, resulting in a total of 120 soil samples.”

(9) Lines 351-352: Replace "harvested" with "collected."

We have replaced “harvested” with “collected” as suggested.

(10) Line 367: Clarify how Illumina adapters and indices were added (e.g., two-step PCR, fusion primers).

We thank the reviewer for this helpful suggestion. We have revised the Methods section to clarify how Illumina adapters and indices were incorporated. We used a pooled amplicon library preparation strategy. Individual samples were first amplified with primers containing sample-specific barcode sequences. The barcoded amplicons from multiple samples were then pooled and used for library preparation with the ALFA-SEQ DNA Library Prep Kit. Universal Illumina-compatible adapters were first ligated to the pooled amplicons. After bead-based purification, an indexing PCR was performed using the index primer mix, which introduced the complete P5/P7 sequences and a library-level Illumina index into the library molecules. Thus, sample demultiplexing was based on the sample-specific barcodes introduced during amplicon PCR, whereas the Illumina index was used to identify the pooled sequencing library. We have clarified this procedure in the revised manuscript.

L424-440

“The community profiles of the GAPDH F-tagged 16S rRNA gene amplicon fragments were determined using high-throughput amplicon sequencing. Briefly, GAPDH F-tagged 16S rRNA gene amplicon fragments from the microcosm soils were first amplified from individual samples using GAPDH F and barcode-labeled 806R primers. The reverse primer 806R carried a 12-bp sample-specific barcode, whereas the GAPDH F primer did not contain a barcode. Therefore, each sample was assigned a unique barcode during PCR, which allowed sample demultiplexing after sequencing. The PCR reaction system and thermal cycling conditions were similar to those described above, except that the number of amplification cycles was increased to 35 to obtain sufficient amplicon products for sequencing. The barcoded PCR products from individual samples were purified using a GeneJET Gel Extraction Kit (Thermo Scientific, Lithuania), quantified, and then pooled in equimolar amounts for subsequent library construction. Sequencing libraries were prepared from the pooled barcoded amplicons using the ALFA-SEQ DNA Library Prep Kit according to the manufacturer’s protocol. Universal Illumina-compatible adapters were first ligated to the pooled amplicon products, followed by bead-based purification. An indexing PCR was then performed using the index primer mix, which introduced the complete P5/P7 flow-cell binding sequences and a library-level Illumina index into the pooled library molecules. The indexed library was purified, quantified, and subjected to paired-end sequencing on the NovaSeq platform at MAGIGENE Co., Ltd. (Guangzhou, China).

(11) Provide more detail on chimera removal, filtering thresholds, and normalization choices.

We thank the reviewer for this helpful suggestion. The raw paired-end reads were first merged, and primer sequences were removed using the search_pcr2 script in USEARCH. Reads with more than two primer mismatches were discarded. Quality filtering was then performed using fastq_filter, and sequences with quality scores below 20 were removed. Redundant reads were collapsed using fastx_uniques. Amplicon sequence variants (ASVs) were generated using the UNOISE3 denoising algorithm, which also performs built-in chimaera filtering during ASV inference. In addition, ASVs with total sequence counts fewer than 9 were excluded to reduce the influence of low-frequency noise.

L443-460

“Briefly, paired-end reads were merged using USEARCH, and primer sequences (GAPDH-F-515F and 806R) were removed using the search_pcr2 script. Reads with more than two primer mismatches were discarded. Quality filtering was performed using the fastq_filter script, and sequences with quality scores below 20 were removed. Redundant sequences were dereplicated using the fastx_uniques script. ASVs were generated using the UNOISE3 non‑clustering denoising algorithm (Edgar, 2016), which infers 100% exact sequence variants by distinguishing biological sequences from PCR/sequencing errors. ASVs with total sequence counts fewer than 9 across all samples were removed to reduce noise. To quantify the abundance of each ASV, an ASV table was generated by mapping the quality‑filtered raw reads back to the ASV set using the otutab command. A 97% similarity threshold was applied for this recruitment to accommodate stochastic sequencing noise while maintaining biological resolution. Crucially, the mapping followed a best-hit priority rule, where each read was assigned to the ASV with the highest per cent identity within the 97% radius. This approach ensures that reads derived from the same biological template are accurately counted toward their respective ASV, preventing the underestimation of abundances that would occur with exact matching while strictly preserving the single-nucleotide resolution of the ASV framework. Taxonomic annotation of the ASVs was performed in QIIME2 with the Silva v138 database. A total of 89322 prokaryotic ASVs were obtained. To standardize sequencing depth across samples, the read number of each sample was rarefied to 53251 using the rarefy function in the vegan package in R.”

(12) Line 412: Rephrase to refer to 16S amplicon addition rather than 16S rRNA genes (along the whole text), as only the V4 region is analyzed.

We thank the reviewer for this helpful suggestion. we have rephrased references to “16S rRNA genes” to “16S rRNA gene amplicon fragments”

(13) Ensure consistent primer naming throughout (e.g., GAPDH F vs ACTF).

We have checked the entire manuscript and confirm that only “GAPDH F” is used as the label primer.

(14) Finally, the manuscript would benefit from careful language editing. Several typographical errors, grammatical inconsistencies, and unclear phrases are present throughout. Examples include:

Misspellings such as "diffrence" (e.g., figure legends) and inconsistent capitalization. Inconsistent terminology (e.g., "genes," "amplicons," and "fragments" used interchangeably without clarification). Redundant or awkward phrasing (e.g., repeated use of "extracellular 16S rRNA genes"). Occasional subject-verb agreement issues and missing articles.

We sincerely apologize for the language issues. The manuscript has now undergone a thorough language editing process by a native English‑speaking colleague.

Recommendation

Major revision: The manuscript addresses an important problem and presents a promising approach. However, key issues related to conceptual clarity, bioinformatic consistency, statistical rigor, and interpretation of PMA-based results must be resolved. With substantial revision and clarification, the study has the potential to make a meaningful contribution to the field.

We sincerely thank the reviewer for the thorough, constructive, and critical evaluation of our manuscript. We greatly appreciate the recognition that our study addresses an important problem and presents a promising approach. We also acknowledge the key issues raised regarding conceptual clarity, bioinformatic consistency, statistical rigor, and interpretation of PMA‑based results. We have taken these comments very seriously and have substantially revised the manuscript accordingly, more details about the revisions are described in the following point-by-point responses.

**Reviewer #2 (Recommendations for the authors):**

Editorial comments:

(1) Title: I recommend removing "across China" from the title. In many ways, the study has nothing to do specifically with China, and you limit the broad applicability of the study. The same work could have been done with soils from Africa, for example. Also, it might be ok to remove 16S rRNA as well. The 16S rRNA genes are a proxy for rates of extracellular DNA degradation, but the study isn't exactly about 16S either.

We thank the reviewer for this thoughtful suggestion regarding the title. We have revised the title to “The overall and sequence-specific degradation of soil extracellular DNA fragments: rates and influential factors.”

(3) L44-45: "...such as real-time PCR, high-throughput amplicon sequencing, and metagenomic analysis...".

We thank the reviewer for this suggestion. We have revised the order according to the suggestions of the reviewer.

L44-45

“The investigation of soil microbial abundance and diversity heavily relies on DNA-based technologies, such as real-time PCR, high-throughput amplicon sequencing, and metagenomic analysis.”

(4) L48: remove "they".

We agree with the reviewer and have removed the extraneous “they”.

(5) L51: "noise factor"; "...persistence can lead to...".

We have revised the sentence as suggested.

(6) L53: remove theoretical.

We have removed “theoretical”.

(7) L58: remove "the".

We have removed "the".

(8) L86: Is restriction digestion of DNA a likely extracellular process in soil?

We thank the reviewer for this thoughtful comment. We agree that the original wording may have overstated the likelihood of classical restriction digestion as a dominant extracellular process in soils. Our intention was not to suggest that intracellular restriction enzyme systems operate directly in the soil matrix in the same manner as they do within living cells. Rather, we aimed to indicate more generally that sequence-dependent nuclease susceptibility could contribute to differential degradation among extracellular DNA fragments.

L87-95

“First, sequence-dependent degradation can arise from differences in nucleotide composition, particularly GC content. This influences the thermodynamic stability and base-stacking interactions of the DNA duplex, thereby altering its accessibility to extracellular nucleases (Marrone and Ballantyne, 2008; Wolpe and Guertin, 2022). Second, local conformational features and the formation of potential secondary structures, such as stem-loops or hairpins, can create steric hindrance that protects the phosphodiester backbone. Differences in base composition also alter the elemental stoichiometry (e.g., C: N ratio) of DNA molecules, potentially affecting microbial preference for recycling specific sequences as nutrient sources (Cai et al., 2006a; Buitrago et al., 2021).”

(9) L94-99: The authors might also consider the different nitrogen content of different bases; this might also affect sequence-specific selection of DNA for degradation.

We thank the reviewer for this insightful suggestion. We agree that differences in the elemental composition of DNA bases, including nitrogen content, may provide an additional mechanistic explanation for sequence-dependent degradation. In the revised manuscript, we have incorporated this point into the Introduction.

L92-95

“Differences in base composition also alter the elemental stoichiometry (e.g., C:N ratio) of DNA molecules, potentially affecting microbial preference for recycling specific sequences as nutrient sources (Cai et al., 2006a; Buitrago et al., 2021).”

(10) L110-112: These are not really written in hypothesis form. Also, what about a hypothesis about degradation rates and soil type/temperature/moisture?

We thank the reviewer for this constructive critique. We have rewritten the hypotheses. To improve the logical flow of the manuscript, we have restructured the Introduction by moving the three central hypotheses immediately following the discussion of the biochemical mechanisms underlying sequence-specific degradation. This adjustment ensures that the hypotheses are directly grounded in the theoretical framework.

L99-104

“Consequently, we proposed three central hypotheses. (1) The degradation rates of eDNA amplicon fragments were expected to be highly sequence‑specific. (2) The rates and patterns of eDNA fragments degradation would be influenced by environmental factors such as temperature and moisture content. (3) The sequence‑specific degradation of extracellular 16S rRNA gene amplicon fragments would significantly influence estimates of soil prokaryotic abundance and diversity.”

(11) L116: "GAPDH F-labeled 16S rRNA gene amplicon fragments....".

We thank the reviewer for this helpful suggestion. we have rephrased references to “16S rRNA genes” to “16S rRNA gene amplicon fragments”

(12) L117: "rapidly".

We agree with the reviewer and have revised.

(13) L118-120: "After a 48-day incubation period, 0.2 to 3.1% of the initial spike GADPH F-labeled 16S rRNA gene amplicon fragments ...".

We agree with the reviewer and have revised.

(14) L125: Spell out SEM in first usage.

We thank the reviewer for this suggestion. In the revised manuscript, we have spelled out SEM as Structural equation modeling.

(15) L128: I don't like the idea of putting this Figure in supplemental materials.

We thank the reviewer for this suggestion. We have moved Figure S2 (moisture gradient microcosm experiment) to the main text as Figure 1f.

(16) L154: The term "intracellular prokaryotic abundance" is not the right term. This makes one think of an intracellular parasite. I think you want something like: "Approximately 40% of sequences in total soil DNA extraction NGS amplicon libraries were derived from intact cells, while the remaining represented extracellular DNA. Conversely, greater than 80% of observed richness was derived from intact cells." (Please check that I stated this correctly.) I would also suggest some statistics or ranges here.

We thank the reviewer for this important terminological clarification. We agree that the term “intracellular prokaryotic abundance” is misleading, as it could imply intracellular parasites. In the revised manuscript, we have replaced this with a clearer description and We have also added the across‑site ranges to provide statistical context.

L163-166

“The PMA treatment revealed that intact cells accounted for approximately 40% (range: 9–73%) of the total 16S rRNA gene copies. In contrast, over 80% (range: 27–97%) of the observed ASV richness was associated with sequences originating from intact cells (Fig. 4a and b).”

(17) L168: "...a significant NEGATIVE correlation was observed...".

We agree with the reviewer and have revised.

(18) L169: "However, no significant relationship was observed...".

We agree with the reviewer and have revised.

(19) L194-195: What about pH and temperature?

We thank the reviewer for this comment. We agree that pH and temperature are important environmental factors that can influence microbial DNA degradation and community composition. However, our results (Fig. 1c) indicate that soil moisture is the most dominant factor affecting extracellular DNA degradation. Therefore, in the revised manuscript, we have focused the explanation primarily on soil moisture, while acknowledging that pH and temperature may also be important influencing factors.

L208-211

“Third, environmental factors, including soil moisture, pH, and temperature, can predominantly govern enzymatic reaction rates (He et al., 2024; Shah et al., 2024). Indeed, strong positive correlations were observed between moisture content and eDNA degradation rates in both the survey and microcosm experiments (Fig. 1d-f).”

(20) L199: "findings".

We have revised as suggested.

(21) L227-229: This sounds more like results.

We thank the reviewer for this comment. We agree that the original first sentence in L227–229 reads more like results. Our intention was to introduce the discussion by linking extracellular DNA to potential impacts on prokaryotic community analysis, rather than to present specific findings at this point. We have reorganized this section as follows.

L246-248

“Accordingly, we further explored how DNA may influence prokaryotic community analyses using PMA treatment, and significant disparities were observed between the profiles of the total and PMA-treated soil prokaryotic communities (Fig. 4).”

(22) L230: Need to also consider differential cell lysis during DNA extraction.

We thank the reviewer for this important comment. We agree that differential cell lysis during DNA extraction could influence the observed community profiles, as microbial taxa differ in cell wall composition and resistance to mechanical or chemical lysis. In the revised manuscript, we explicitly acknowledge this limitation in the relevant section. We also clarify that a standardized DNA extraction protocol (DNeasy PowerSoil kit) was used to efficiently lyse a broad range of microbial taxa, but some taxon-specific lysis bias may remain. Future studies could combine multiple lysis methods or spike-in controls to quantify and correct for potential extraction bias.

L262-265

“However, as DNA extraction efficiency may differ between intact cells and eDNA, the actual differences between total and living prokaryotic abundance could be smaller than those observed in this study. Similarly, the overestimated prokaryotic richness may arise from historically accumulated microbial taxonomic information stored in eDNA pools (Deshpande and Fahrenfeld, 2023; Wang et al., 2024).”

L309-311

“Compounding this issue, downstream DNA recovery is subject to differential cell lysis, as taxa with robust cell walls (e.g., Gram-positive bacteria) may resist extraction (Frostegård et al., 1999; Albertsen et al., 2015).”

(23) L232: Need to also consider that PMA treatment is not perfect and can be affected by substrate, the ability of light to access DNA for crosslinking, etc.

We thank the reviewer for this important reminder. We agree that PMA treatment is not perfect and that its efficiency can be affected by soil matrix properties (e.g., organic matter, clay minerals) and the ability of light to penetrate the sample for DNA crosslinking. In the revised manuscript, we have explicitly acknowledged these limitations in the discussion.

L305-314

“Second, methodological biases inherent in quantifying the intracellular community must be acknowledged (Du et al., 2025). Although PMA treatment is widely used to exclude eDNA, its efficiency in complex soil matrices can be compromised by limited light penetration in turbid suspensions and competitive adsorption to soil particles (Nocker et al., 2007; Carini et al., 2016; Heise et al., 2016). Compounding this issue, downstream DNA recovery is subject to differential cell lysis, as taxa with robust cell walls (e.g., Gram-positive bacteria) may resist extraction (Frostegård et al., 1999; Albertsen et al., 2015). While our standardized bead-beating protocol and calculation of degradation rate constants (*k*) minimize systematic biases, future studies should integrate complementary viability markers (e.g., RNA-based analyses or protein synthesis activity probes) and multi-extraction comparisons to robustly validate these ecological patterns (Emerson et al., 2017).”

(24) L240: Can extracellular DNA have an ecological role?

We thank the reviewer for this thoughtful question. Yes, extracellular DNA (eDNA) does have important ecological roles beyond being a potential bias in molecular analyses. In the revised manuscript, we have added statements to highlight that extracellular DNA can serve as a nutrient source (e.g., nitrogen and phosphorus) for microbes and may also contribute to horizontal gene transfer. This emphasizes that extracellular DNA may actively influence microbial community structure and function, in addition to its role in potentially inflating observed abundance and richness.

L267-275

“We observed a significant correlation between eDNA degradation rates and the overall structure of the prokaryotic community, but this relationship was absent in PMA-treated communities (Fig. 5b). This discrepancy highlights the divergent ecological roles of extracellular and intracellular DNA. Analyses of the total community integrate intracellular DNA from metabolically active cells with eDNA which primarily originates from historical microbial residues (Lennon et al., 2018). EDNA incorporates signals that likely reflect the legacy effects of past environmental conditions (Wang et al., 2021). In contrast, the PMA-treated community reflects transient microbial activity driven by current selective pressures. Additionally, eDNA can serve as a nutrient source and facilitate horizontal gene transfer, which may further shape its interactions with contemporary microbial communities (Levy-Booth et al., 2007).”

(25) L256: Why would microorganisms selectively degrade one DNA sequence vs another? This seems to be likely to be stochastic in terms of which sequences are taken up by microorganisms. However, different DNA sequences might hydrolyze differently or be otherwise damaged, and that could lead to differential degradation of a viable amplicon. It might be interesting to incorporate long pieces of DNA with different internal primer sites and use quantitative PCR to determine how sequences are degrading.

We thank the reviewer for this important mechanistic insight. We agree that the observed correlation between degradation rate and sequence abundance does not necessarily imply active microbial preference. It could equally reflect stochastic encounter rates or intrinsic chemical differences (e.g., AT‑rich regions hydrolyzing faster). We have revised the corresponding paragraph in the Discussion.

L279-292

“This finding suggests that abundant eDNA degrades at a faster rate compared to rare eDNA. As mentioned earlier, this could be explained by several mechanisms. First, as soil eDNA is subject to enzymatic degradation and microbial recycling, abundant DNA sequences may be more likely to be encountered and degraded by extracellular nucleases simply due to their higher copy numbers (Levy-Booth et al., 2007; Nagler et al., 2018). Similarly, if microbes preferentially take up DNA as a nutrient source, they may degrade abundant sequences more frequently as a stochastic consequence of higher encounter rates (Finkel and Kolter, 2001). However, we also found that the relationships between the sequence-specific degradation rates and the effect sizes of extracellular 16S rRNA gene amplicon fragments varied across the study sites (Fig. S1g). The sequence-specific effect sizes of extracellular 16S rRNA gene amplicon fragments are mainly determined by both their production and degradation rates (Pietramellara et al., 2009; Sirois and Buckley, 2019). These inconsistent correlations emphasize the critical role played by the production rates of extracellular 16S rRNA genes in influencing the analysis of prokaryotic communities. Therefore, future studies should systematically determine both the production and degradation rates of eDNA.”

(26) L282-283: This belongs in the discussion.

We agree with the reviewer and have revised accordingly.

(27) L289: "as well as measurements of total organic carbon".

We agree with the reviewer and have revised accordingly.

(28) L338: Any water content for these soils?

We thank the reviewer for this comment. The water contents of soils from all study sites are reported in Supplementary Table 2.

(29) L349-350: You mean that you measured the total soil extracted DNA and then added 1% as labeled 16S?

Yes, for each soil sample, we extracted total soil DNA and quantified its concentration (ng DNA per gram of soil). We then added exogenous GAPDH‑tagged 16S amplicon fragments at an amount equal to 1% of this total DNA concentration. This concentration was chosen to mimic a realistic pulse of extracellular DNA input without overwhelming the endogenous DNA pool. We apologize for any confusion caused by the imprecise wording in the original manuscript.

L392-398

“The microcosm experiment was conducted using 30 g of soil for each sample. After pre-incubation at 20℃ for one week, each soil was thoroughly mixed with the GAPDH F‑tagged 16S rRNA gene amplicon fragments and incubated further at 20℃ (Fig. S8). The amount of exogenous GAPDH F‑tagged 16S rRNA gene amplicon fragments added to each soil sample was equivalent to 1% of the total DNA concentration naturally present in that soil, as determined fluorometrically prior to the experiment. This concentration was chosen to approximate natural eDNA fluxes resulting from microbial lysis, ensuring experimental relevance to in situ conditions (Table S2).”

(30) L354: Remember that soil recovery from intact cells is going to be lower than for extracellular DNA. So, you are probably overestimating the contribution of extracellular DNA to the total DNA in the system.

We thank the reviewer for this comment. We agree that DNA recovery from intact cells is generally lower than from extracellular DNA due to differential cell lysis efficiencies. Consequently, the contribution of extracellular DNA to total soil DNA may be somewhat overestimated in our study. We have clarified this limitation in the revised manuscript.

L262-265

“However, as DNA extraction efficiency may differ between intact cells and eDNA, the actual differences between total and living prokaryotic abundance could be smaller than those observed in this study. Similarly, the overestimated prokaryotic richness may arise from historically accumulated microbial taxonomic information stored in eDNA pools.”

L305-314

“Second, methodological biases inherent in quantifying the intracellular community must be acknowledged (Du et al., 2025). Although PMA treatment is widely used to exclude eDNA, its efficiency in complex soil matrices can be compromised by limited light penetration in turbid suspensions and competitive adsorption to soil particles (Nocker et al., 2007; Carini et al., 2016; Heise et al., 2016). Compounding this issue, downstream DNA recovery is subject to differential cell lysis, as taxa with robust cell walls (e.g., Gram-positive bacteria) may resist extraction (Frostegård et al., 1999; Albertsen et al., 2015). While our standardized bead-beating protocol and calculation of degradation rate constants (*k*) minimize systematic biases, future studies should integrate complementary viability markers (e.g., RNA-based analyses or protein synthesis activity probes) and multi-extraction comparisons to robustly validate these ecological patterns (Emerson et al., 2017).”

(31) L362: Amplification efficiency is pretty low. I think you would have been better served with GAPDH on both ends, and that would have given you a much higher efficiency qPCR.

We thank the reviewer for this comment. The actual qPCR amplification efficiency in our assay was approximately 85%, which, although slightly below the ideal range, was still acceptable and produced reproducible amplification curves and reliable quantification for degradation-rate calculations.

We acknowledge that the amplification efficiency in our qPCR experiments using a GAPDH F-labeled 16S primer on one end was suboptimal. The current design used a single GAPDH tag at the forward primer to avoid potential amplification bias or primer-dimer formation that could arise from extending the degenerate reverse primer. In addition, dual-end labeling would have required synthesis of new barcode-labeled tagged primers, increasing both cost and experimental complexity. Thanks again for the constructive comments, which provided us with the direction for future experiment optimization.

L365-371

“The GAPDH was incorporated only into the forward primer for several reasons. Methodologically, adding a long linker to the degenerate reverse primer (806R) could reduce amplification efficiency or introduce bias. Economically, single-end labeling allowed us to use the standard reverse primer already carrying sample-specific barcodes, avoiding the costly synthesis of a full set of dual-labeled barcoded primers. This design minimized the risk of secondary structure and primer-dimer artifacts while maintaining sufficient specificity and compatibility with downstream qPCR and sequencing.”

(32) L367: Not enough detail on how barcoded libraries were made. UDIs?

We thank the reviewer for this helpful comment. We have now clarified the library preparation and indexing strategy in the revised Methods section. This amplicon diversity sequencing used a pooled-library strategy. Individual samples were first distinguished by sample-specific inline barcodes introduced during the amplicon PCR step. After amplification, barcoded PCR products from multiple samples were pooled and subjected to library construction using the ALFA-SEQ DNA Library Prep Kit. Universal Illumina-compatible adapters were ligated to the pooled amplicons, followed by an indexing PCR that introduced the complete P5/P7 sequences and a library-level Illumina index. Thus, the Illumina index was used to identify the pooled sequencing library, whereas sample demultiplexing was performed according to the sample-specific inline barcodes. We have revised the Methods section to make this procedure explicit.

L424-440

“The community profiles of the GAPDH F-tagged 16S rRNA gene amplicon fragments were determined using high-throughput amplicon sequencing. Briefly, GAPDH F-tagged 16S rRNA gene amplicon fragments from the microcosm soils were first amplified from individual samples using GAPDH F and barcode-labeled 806R primers. The reverse primer 806R carried a 12-bp sample-specific barcode, whereas the GAPDH F primer did not contain a barcode. Therefore, each sample was assigned a unique barcode during PCR, which allowed sample demultiplexing after sequencing. The PCR reaction system and thermal cycling conditions were similar to those described above, except that the number of amplification cycles was increased to 35 to obtain sufficient amplicon products for sequencing. The barcoded PCR products from individual samples were purified using a GeneJET Gel Extraction Kit (Thermo Scientific, Lithuania), quantified, and then pooled in equimolar amounts for subsequent library construction. Sequencing libraries were prepared from the pooled barcoded amplicons using the ALFA-SEQ DNA Library Prep Kit according to the manufacturer’s protocol. Universal Illumina-compatible adapters were first ligated to the pooled amplicon products, followed by bead-based purification. An indexing PCR was then performed using the index primer mix, which introduced the complete P5/P7 flow-cell binding sequences and a library-level Illumina index into the pooled library molecules. The indexed library was purified, quantified, and subjected to paired-end sequencing on the NovaSeq platform at MAGIGENE Co., Ltd. (Guangzhou, China).”

(33) L368: Why was the # of cycles increased?

Thank you for your question. In the original manuscript (L368), we stated that the number of PCR cycles was increased to 35. This was mainly because the exogenously added GAPDH F‑labeled 16S rRNA genes had a relatively low initial abundance in the soil and gradually degraded during the microcosm incubation, with their copy numbers becoming particularly low at the last time points (see Fig. 1a). To ensure sufficient PCR product for high‑throughput sequencing from samples at all time points (especially those with low abundance at later stages), we appropriately increased the cycle number to 35.

L429-431

“The PCR reaction system and thermal cycling conditions were similar to those described above, except that the number of amplification cycles was increased to 35 to obtain sufficient amplicon products for sequencing.”

(34) L372: Were sequencing adapters ligated onto the pool?

We thank the reviewer for this question. Yes, in this amplicon diversity sequencing workflow, sequencing adapters were ligated onto the pooled amplicon products. Briefly, individual samples were first amplified with sample-specific barcode sequences, allowing each sample to be distinguished after sequencing. The barcoded PCR products from multiple samples were then pooled for library construction. Universal Illumina-compatible adapters were ligated to this pooled amplicon library using the ALFA-SEQ DNA Library Prep Kit. After adapter ligation and purification, an indexing PCR was performed to introduce the complete P5/P7 sequences and a library-level Illumina index. We have clarified this pooled-library construction workflow in the revised Methods section.

L424-440

“The community profiles of the GAPDH F-tagged 16S rRNA gene amplicon fragments were determined using high-throughput amplicon sequencing. Briefly, GAPDH F-tagged 16S rRNA gene amplicon fragments from the microcosm soils were first amplified from individual samples using GAPDH F and barcode-labeled 806R primers. The reverse primer 806R carried a 12-bp sample-specific barcode, whereas the GAPDH F primer did not contain a barcode. Therefore, each sample was assigned a unique barcode during PCR, which allowed sample demultiplexing after sequencing. The PCR reaction system and thermal cycling conditions were similar to those described above, except that the number of amplification cycles was increased to 35 to obtain sufficient amplicon products for sequencing. The barcoded PCR products from individual samples were purified using a GeneJET Gel Extraction Kit (Thermo Scientific, Lithuania), quantified, and then pooled in equimolar amounts for subsequent library construction. Sequencing libraries were prepared from the pooled barcoded amplicons using the ALFA-SEQ DNA Library Prep Kit according to the manufacturer’s protocol. Universal Illumina-compatible adapters were first ligated to the pooled amplicon products, followed by bead-based purification. An indexing PCR was then performed using the index primer mix, which introduced the complete P5/P7 flow-cell binding sequences and a library-level Illumina index into the pooled library molecules. The indexed library was purified, quantified, and subjected to paired-end sequencing on the NovaSeq platform at MAGIGENE Co., Ltd. (Guangzhou, China).”

(35) L378: "Amplicon sequence variants".

We agree with the reviewer and have revised accordingly.

(36) L380: Why were ASVs with fewer than 9 reads removed?

We thank the reviewer for this question. The threshold of removing ASVs with fewer than 9 total reads across all samples was applied to reduce noise from sequencing errors and PCR artifacts. Our justification is supported by both the default parameters of the UNOISE3 algorithm and common practice in amplicon sequencing analysis.

The USEARCH manual specifies that the -minsize parameter in the unoise3 command defaults to 8. This means that unique sequences occurring fewer than 8 times are discarded by the algorithm during ASV inference, as they are unlikely to represent true biological variants. Our threshold of 9 is slightly more conservative than the default (9 > 8), ensuring that only ASVs with a minimal level of abundance are retained. This choice is directly aligned with the algorithm’s intrinsic noise‑filtering logic.

(37) L402: Please don't forget to discuss that PCR bias can contribute to uncertainty in the abundance of each taxon.

Thank you for this important reminder. We agree that PCR bias (e.g., primer‑template mismatches, GC content differences, and variable amplification efficiency) can contribute to uncertainty in the abundance estimates of each taxon. Following your suggestion, we have now added a paragraph in the Discussion section to address this issue. We state that sequence‑specific degradation rates and PCR bias may jointly affect the accuracy of taxon abundance estimates, and future studies should incorporate internal standards or multiplex PCR strategies to correct for such biases. Thank you for your careful review.

L294-305

“Despite the high-resolution insights afforded by our methodology, several limitations should be considered. First, utilizing PCR-amplified 16S rRNA gene fragments as proxies oversimplifies the structural and sequence complexity of natural soil eDNA pools. In natural environments, eDNA varies widely in fragment length and conformation, and exhibits complex interactions with mineral surfaces, all of which fundamentally affect degradation dynamics (Levy-Booth et al., 2007; McKinney and Dungan, 2020). Additionally, the highly conserved nature of the 16S rRNA gene means that the nucleotide variability explored here (e.g., GC content gradients) does not fully capture the genomic heterogeneity of entire metagenomes (Knight et al., 2018). Consequently, our reported degradation rates indicate the decay potential of highly accessible linear eDNA rather than a universal rate for all soil DNA fractions. Future studies incorporating diverse metagenomic DNA, especially those with extreme AT or GC contents, are essential for building a more generalizable predictive framework for eDNA persistence (Morrissey et al., 2015).”

(38) L414: Suggest: "To inhibit amplification of extracellular DNA, soils were incubated with propidium monoazide (PMA), as described previously (REF). Briefly, soil (X grams) was mixed with PMA in a total volume of Y (ml).

We thank the reviewer for this suggestion. We have revised the Methods section to provide a clearer description of PMA treatment, specifying the soil amount (0.50 g) and the total volume (0.5 mL).

L496-497

“To inhibit amplification of eDNA, soils were incubated with PMA, as described previously (Carini et al., 2016).”

L505-506

“In this study, 0.50 g of soil was mixed with PMA in a total volume of 0.5 mL (40 µM PMA in phosphate‑buffered saline, PBS), while the control soil samples were mixed with PBS without PMA.”

(39) L416: In contrast, microbes with intact cell membranes exclude PMA, and their DNA is not cross-linked with PMA, and remains amenable to PCR amplification.

We agree and have revised.

(40) L418-420: wording/sentence is strange and needs work.

Thank you for pointing this out. We have reviewed the sentence at L418‑420 and agree that the wording is awkward. Moreover, the content only listed the advantages of the PMA method without acknowledging its limitations, making the statement less balanced. Therefore, in the revised manuscript, we have deleted this sentence. The limitations of the PMA method have been addressed in the Discussion section.

L502-503

“Currently, PMA treatment is a widely used to suppress PCR amplification of eDNA (Xue et al., 2023; Canini et al., 2024).”

L305-314

“Second, methodological biases inherent in quantifying the intracellular community must be acknowledged (Du et al., 2025). Although PMA treatment is widely used to exclude eDNA, its efficiency in complex soil matrices can be compromised by limited light penetration in turbid suspensions and competitive adsorption to soil particles (Nocker et al., 2007; Carini et al., 2016; Heise et al., 2016). Compounding this issue, downstream DNA recovery is subject to differential cell lysis, as taxa with robust cell walls (e.g., Gram-positive bacteria) may resist extraction (Frostegård et al., 1999; Albertsen et al., 2015). While our standardized bead-beating protocol and calculation of degradation rate constants (*k*) minimize systematic biases, future studies should integrate complementary viability markers (e.g., RNA-based analyses or protein synthesis activity probes) and multi-extraction comparisons to robustly validate these ecological patterns (Emerson et al., 2017).”

(41) L421-422: PMA treatment is a widely used method for inhibiting the enzymatic processing of extracellular DNA (Xue, Canini).

We agree and have revised.

(42) L425: include volume of PBA.

We thank the reviewer for this comment. We have revised the Methods section to include the volume of PMA used

L505-506

“In this study, 0.50 g of soil was mixed with PMA in a total volume of 0.5 mL (40 µM PMA in phosphate‑buffered saline, PBS).”

(43) L429-430: Don't use the word precipitates- use "pellets".

We agree and have revised.

(44) L433: "The abundance of 16S rRNA genes was determined using quantitative PCR employing a LightCycler...".

We agree and have revised.

(45) L445-: Section 4.9 - needs citations for PERMANOVA, NMDS, SEM, etc.

Thank you for your suggestion. We have added the necessary citations for PERMANOVA, NMDS, SEM, and other methods in Section 4.9.

L531-539

Prokaryotic community structure differences among the study sites and incubation time points were examined through non-metric multidimensional scaling analysis (NMDS), permutation multivariate analysis of variance (PERMANOVA), and Permutational Analysis of Multivariate Dispersion (PERMDISP) (Kruskal, 1964; Anderson, 2001). Random forest modeling was conducted to assess the importance of environmental and soil variables in predicting the overall degradation rates of extracellular 16S rRNA gene amplicon fragments. Structural equation modeling (SEM) was employed to further evaluate the direct and indirect effects of soil moisture, soil pH, MAP, and prokaryotic abundance on the overall degradation rates of extracellular 16S rRNA gene amplicon fragments (Grace, 2006).

(46) L698: A few comments. It would be nice to know how many different 16S sequences were tracked for differential degradation and shown in the figure.

We thank the reviewer for this helpful comment. We would like to clarify that Fig. 1A does not track the degradation of individual 16S rRNA gene amplicon sequences, but instead shows the overall degradation dynamics of the total added exogenous DNA pool. The data points are derived from total 16S gene copy numbers measured via qPCR at each incubation time point. Consequently, this quantification inherently includes all sequences present within the added pool. The multiple lines visualized in the figure represent the collective degradation trajectories of the entire DNA pool across different study sites

To address sequence-level changes, we further analyzed the richness and composition of the GAPDH F-tagged 16S rRNA gene amplicon fragments, which are presented in Fig. 2A and related analyses.

(47) L699: Better to use "16S rRNA gene amplicon fragment abundance" as the term.

We thank the reviewer for this helpful suggestion. In the revised manuscript, we have replaced the original wording with “16S rRNA gene amplicon fragment abundance” where appropriate.

(48) Y-axis for Figures 1A and 2A should be GAPDH-labeled, not ACTB-labeled.

We apologize for this mistake. We have corrected this error in the revised manuscript.

(49) For Figure 1b: Why not use box plots and ANOVA for different soil types?

Thank you for your valuable suggestion. In the original Figure 1b, we used a bar plot to display the degradation rate constants across the 30 study sites. This choice was intended to emphasize the continuous variation among sites and their gradient relationships with environmental factors (e.g., soil moisture, MAP), which were then used in random forest and structural equation modeling. The bar plot better illustrates the spatial continuum of degradation rates rather than treating ecosystem types as discrete categories.

Nevertheless, we fully agree that a boxplot grouped by ecosystem type (grassland, forest, cropland, desert) would help readers quickly grasp the overall differences among land‑use types. In the revised manuscript, we have added a boxplot grouped by ecosystem type and performed one‑way ANOVA followed by Tukey HSD post‑hoc tests (Fig. 1.). The results show that degradation rate constants differ significantly among ecosystem types (P < 0.05).

L124-128

“The degradation rate constants of the spiked extracellular 16S rRNA gene amplicon fragments displayed considerable variability among the study sites, ranging from 0.05 to 0.16 day^-1^ (Fig. 1b). Furthermore, we found that degradation rate constants differed significantly among ecosystem types (Fig. 1c, *P* < 0.05). Specifically, cropland and forest soils exhibited significantly higher degradation rates than grassland soils (*P* < 0.05).”

(50) For Figure 2: Where are PERMANOVA and PERMDISP values for the figure?

We thank the reviewer for this comment. In the revised manuscript, we have added the PERMANOVA and PERMDISP values corresponding to Figure 2 in the figure legend and Results section (Fig. 2).

(51) I found Figure 2b to be hard to see. The 48-day circles are almost invisible. Difficult to know what the authors are trying to show here, since there is so much variability associated with soil type.

We thank the reviewer for this comment. Figure 2b is intended to illustrate the temporal changes in microbial community structure during the incubation. The different colored circles represent samples at different time points (1, 3, 6, 12, 24, and 48 days), showing how communities shift over time. We apologize that in the original Figure 2b, the 48‑day samples were nearly invisible and that the high variability among soil types obscured the intended message. In the revised manuscript, we have added a black border around every data point, which greatly enhances the visibility of the 48‑day samples (and all time points). We now use distinct shapes to represent different ecosystem types (grassland, forest, cropland, desert) in the NMDS ordination, and added PERMANOVA results in both the Results section and the figure legend (Fig. R2b).

(52) Figure 4A: Y-axis need a label like "16S rRNA gene abundance".

We agree and have revised.

(53) Figure 4B: I'd like to see a Shannon index too, not just richness.

Thank you for your suggestion. We agree that the Shannon index, which integrates both richness and evenness, provides a valuable complement to richness alone. In the revised manuscript, we added an analysis of the Shannon index to compare α‑diversity between total DNA (PMA‑untreated) and intact cell DNA (PMA‑treated) samples (Fig.4).

(54) Figure 4D: Would be good to have lines linking the intact cell vs total abundance. Also, what about a box plot of Bray-Curtis (or similar) dissimilarity between intact cell and total microbial analysis across the dataset?

Thank you for your suggestions. Regarding the addition of connecting lines in Figure 4D, after careful consideration we decided not to add them for the following reason: the total and PMA-treated communities from the same site are already coded with the same color (different colors for different sites), which effectively indicates the pairing. Adding lines would greatly reduce readability due to dense overlapping lines, especially given the number of sites. Therefore, we kept the original color‑based pairing design.

To address your second suggestion, we have added a bar plot showing the distribution of Bray‑Curtis dissimilarities between total (PMA‑untreated) and intact cell (PMA‑treated) communities across all study samples (Fig. R3d).

(55) Figure 5B: What do correlations with p > 0.05 show? I would remove these from the image.

We thank the reviewer for this suggestion. We agree that correlations with p > 0.05 do not represent statistically significant relationships and may cause confusion. In the revised manuscript, we have removed these non-significant correlations from Figure 5B.

(56) Figure 6: "Incubations of 0, 3, 6, 12, 24, and 48 days".

We agree with the reviewer and have revised as suggested.

References

Albertsen, M., Karst, S.M., Ziegler, A.S., Kirkegaard, R.H., Nielsen, P.H., 2015. Back to basics–the influence of DNA extraction and primer choice on phylogenetic analysis of activated sludge communities. PLoS One 10, e0132783.

Anderson, M.J., 2001. A new method for non‐parametric multivariate analysis of variance. Austral Ecology 26, 32-46.

Arvizu-Hernandez, E., Ocadiz-Delgado, R., Gariglio, P., 2025. E7HPV16 Oncogene and 17beta-Estradiol Stress Promote Oncogenic microRNA Expression Patterns, Cell Proliferation and Cervical Intraepithelial Neoplasia 1. Cell Biochemistry and Function 43, e70065.

Buitrago, D., Labrador, M., Arcon, J.P., Lema, R., Flores, O., Esteve-Codina, A., Blanc, J., Villegas, N., Bellido, D., Gut, M., 2021. Impact of DNA methylation on 3D genome structure. Nature Communications 12, 3243.

Cai, P., Huang, Q., Zhang, X., Chen, H., 2006a. Adsorption of DNA on clay minerals and various colloidal particles from an Alfisol. Soil Biology and Biochemistry 38, 471-476.

Cai, P., Huang, Q.Y., Zhang, X.W., 2006b. Interactions of DNA with clay minerals and soil colloidal particles and protection against degradation by DNase. Environmental Science & Technology 40, 2971-2976.

Carini, P., Marsden, P.J., Leff, J.W., Morgan, E.E., Strickland, M.S., Fierer, N., 2016. Relic DNA is abundant in soil and obscures estimates of soil microbial diversity. Nature Microbiology 2, 1-6.

Deshpande, A.S., Fahrenfeld, N.L., 2023. Influence of DNA from non-viable sources on the riverine water and biofilm microbiome, resistome, mobilome, and resistance gene host assignments. Journal of Hazardous materials 446, 130743.

Du, Y., Wang, Z., Liu, K., Chai, G., Chi, Y., Li, T., Duan, Y., Xia, T., Liu, D., Che, R., 2025. The performance of different methods in characterizing soil live prokaryotic diversity and abundance is highly variable. iMetaOmics, e70011.

Emerson, J.B., Adams, R.I., Román, C.M.B., Brooks, B., Coil, D.A., Dahlhausen, K., Ganz, H.H., Hartmann, E.M., Hsu, T., Justice, N.B., 2017. Schrödinger’s microbes: tools for distinguishing the living from the dead in microbial ecosystems. Microbiome 5, 86.

Finkel, S.E., Kolter, R., 2001. DNA as a nutrient: novel role for bacterial competence gene homologs. Journal of Bacteriology 183, 6288-6293.

Frostegård, Å., Courtois, S., Ramisse, V., Clerc, S., Bernillon, D., Le Gall, F., Jeannin, P., Nesme, X., Simonet, P., 1999. Quantification of bias related to the extraction of DNA directly from soils. Applied and Environmental Microbiology 65, 5409-5420.

Grace, J.B., 2006. Structural equation modeling and natural systems. Cambridge University Press.

He, P., Li, L.-J., Dai, S.-S., Guo, X.-L., Nie, M., Yang, X., Kuzyakov, Y., 2024. Straw addition and low soil moisture decreased temperature sensitivity and activation energy of soil organic matter. Geoderma 442, 116802.

Heise, J., Nega, M., Alawi, M., Wagner, D., 2016. Propidium monoazide treatment to distinguish between live and dead methanogens in pure cultures and environmental samples. Journal of Microbiological Methods 121, 11-23.

Huang, C., Xie, D.C., Cui, J.J., Li, Q., Gao, Y., Xie, K.P., 2014. FOXM1c Promotes Pancreatic Cancer Epithelial-to-Mesenchymal Transition and Metastasis via Upregulation of Expression of the Urokinase Plasminogen Activator System. Clinical Cancer Research 20, 1477-1488.

Knight, R., Vrbanac, A., Taylor, B.C., Aksenov, A., Callewaert, C., Debelius, J., Gonzalez, A., Kosciolek, T., McCall, L.-I., McDonald, D., 2018. Best practices for analysing microbiomes. Nature Reviews Microbiology 16, 410-422.

Kruskal, J.B., 1964. Nonmetric multidimensional scaling: a numerical method. Psychometrika 29, 115-129.

Lennon, J.T., Muscarella, M.E., Placella, S.A., Lehmkuhl, B.K., 2018. How, when, and where relic DNA affects microbial diversity. mbio 9, e00637-00618.

Levy-Booth, D.J., Campbell, R.G., Gulden, R.H., Hart, M.M., Powell, J.R., Klironomos, J.N., Pauls, K.P., Swanton, C.J., Trevors, J.T., Dunfield, K.E., 2007. Cycling of extracellular DNA in the soil environment. Soil Biology and Biochemistry 39, 2977-2991.

Liu, Q.H., Yuan, L., Li, Z.H., Leung, K.M.Y., Sheng, G.P., 2024. Natural organic matter enhances natural transformation of extracellular antibiotic resistance genes in sunlit water. Environmental Science & Technology 58, 17990-17998.

Marrone, A., Ballantyne, J., 2008. Sequence Specificity of BAL 31 Nuclease for ssDNA Revealed by Synthetic Oligomer Substrates Containing Homopolymeric Guanine Tracts. PLoS One 3, e3595.

McKinney, C.W., Dungan, R.S., 2020. Influence of environmental conditions on extracellular and intracellular antibiotic resistance genes in manure-amended soil: A microcosm study. Soil Science Society of America Journal 84, 747-759.

Morrissey, E.M., McHugh, T.A., Preteska, L., Hayer, M., Dijkstra, P., Hungate, B.A., Schwartz, E., 2015. Dynamics of extracellular DNA decomposition and bacterial community composition in soil. Soil Biology and Biochemistry 86, 42-49.

Nagler, M., Insam, H., Pietramellara, G., Ascher-Jenull, J., 2018. Extracellular DNA in natural environments: features, relevance and applications. Applied Microbiology and Biotechnology 102, 6343-6356.

Nocker, A., Sossa-Fernandez, P., Burr, M.D., Camper, A.K., 2007. Use of propidium monoazide for live/dead distinction in microbial ecology. Applied and Environmental Microbiology 73, 5111-5117.

Pietramellara, G., Ascher, J., Borgogni, F., Ceccherini, M., Guerri, G., Nannipieri, P., 2009. Extracellular DNA in soil and sediment: fate and ecological relevance. Biology and Fertility of Soils 45, 219-235.

Shah, A., Huang, J., Han, T., Khan, M.N., Tadesse, K.A., Daba, N.A., Khan, S., Ullah, S., Sardar, M.F., Fahad, S., 2024. Impact of soil moisture regimes on greenhouse gas emissions, soil microbial biomass, and enzymatic activity in long-term fertilized paddy soil. Environmental Sciences Europe 36, 120.

Sirois, S.H., Buckley, D.H., 2019. Factors governing extracellular DNA degradation dynamics in soil. Environmental Microbiology Reports 11, 173-184.

Vuillemin, A., Horn, F., Alawi, M., Henny, C., Wagner, D., Crowe, S.A., Kallmeyer, J., 2017. Preservation and significance of extracellular DNA in ferruginous sediments from Lake Towuti, Indonesia. Frontiers in Microbiology 8, 1440.

Wang, X., Ganzert, L., Bartholomaus, A., Amen, R., Yang, S., Guzman, C.M., Matus, F., Albornoz, M.F., Aburto, F., Oses-Pedraza, R., Friedl, T., Wagner, D., 2024. The effects of climate and soil depth on living and dead bacterial communities along a longitudinal gradient in Chile. The Science of the total environment 945, 173846.

Wang, Y.-T., Yang, W.-J., Li, C.-L., Doudeva, L.G., Yuan, H.S., 2007. Structural basis for sequence-dependent DNA cleavage by nonspecific endonucleases. Nucleic Acids Research 35, 584-594.

Wang, Y., Yan, Y., Thompson, K.N., Bae, S., Accorsi, E.K., Zhang, Y., Shen, J., Vlamakis, H., Hartmann, E.M., Huttenhower, C., 2021. Whole microbial community viability is not quantitatively reflected by propidium monoazide sequencing approach. Microbiome 9, 1-13.

Wolpe, J., Guertin, M., 2022. Regional and Single Nucleotide Correction of Sequence Bias in Chromatin Accessibility Data. The FASEB Journal 36.

Yang, A., Liu, X., Liu, P., Feng, Y.Z., Liu, H.B., Gao, S., Huo, L.M., Han, X.Y., Wang, J.R., Kong, W., 2021. LncRNA UCA1 promotes development of gastric cancer via the miR-145/MYO6 axis. Cellular & Molecular Biology Letters 26, 33.

Ye, M., Zhang, Z., Sun, M., Shi, Y., 2022. Dynamics, gene transfer, and ecological function of intracellular and extracellular DNA in environmental microbiome. iMeta 1, e34.
